# High-density linkage map construction in an autotetraploid blueberry population and detection of quantitative trait loci for anthocyanin content

**DOI:** 10.3389/fpls.2022.965397

**Published:** 2022-09-23

**Authors:** Sara Montanari, Susan Thomson, Sarah Cordiner, Catrin S. Günther, Poppy Miller, Cecilia H. Deng, Tony McGhie, Mareike Knäbel, Toshi Foster, Janice Turner, David Chagné, Richard Espley

**Affiliations:** ^1^The New Zealand Institute for Plant and Food Research Limited, Motueka, New Zealand; ^2^The New Zealand Institute for Plant and Food Research Limited, Lincoln, New Zealand; ^3^The New Zealand Institute for Plant and Food Research Limited, Palmerston North, New Zealand; ^4^The New Zealand Institute for Plant and Food Research Limited, Ruakura, New Zealand; ^5^The New Zealand Institute for Plant and Food Research Limited, Te Puke, New Zealand; ^6^The New Zealand Institute for Plant and Food Research Limited, Auckland, New Zealand

**Keywords:** *Vaccinium corymbosum*, fruit quality, flavonoids, SNP markers, genetic map, candidate genes

## Abstract

Highbush blueberry (*Vaccinium corymbosum*, 2n = 4x = 48) is the most cultivated type of blueberry, both in New Zealand and overseas. Its perceived nutritional value is conferred by phytonutrients, particularly anthocyanins. Identifying the genetic mechanisms that control the biosynthesis of these metabolites would enable faster development of cultivars with improved fruit qualities. Here, we used recently released tools for genetic mapping in autotetraploids to build a high-density linkage map in highbush blueberry and to detect quantitative trait loci (QTLs) for fruit anthocyanin content. Genotyping was performed by target sequencing, with ∼18,000 single nucleotide polymorphism (SNP) markers being mapped into 12 phased linkage groups (LGs). Fruits were harvested when ripe for two seasons and analyzed with high-performance liquid chromatography-mass spectrometry (HPLC-MS): 25 different anthocyanin compounds were identified and quantified. Two major QTLs that were stable across years were discovered, one on LG2 and one on LG4, and the underlying candidate genes were identified. Interestingly, the presence of anthocyanins containing acylated sugars appeared to be under strong genetic control. Information gained in this study will enable the design of molecular markers for marker-assisted selection and will help build a better understanding of the genetic control of anthocyanin biosynthesis in this crop.

## Introduction

Blueberry is an important soft fruit crop native to North America, only recently domesticated within the twentieth century in New Jersey, US (Ehlenfeldt, 2009). Blueberries belong to the *Cyanococcus* section of the genus *Vaccinium*, which encompasses an estimated 150–450 species, including bilberries, cranberries, and lingonberries (Retamales and Hancock, 2018). Cultivated blueberries are mainly from species of *V. corymbosum* L. (highbush blueberry), *V. virgatum* Ait. (rabbiteye blueberry; syn. *V. ashei* Reade) and native stands of *V. angustifolium* Ait. (lowbush blueberry) (Luby et al., 1991; Retamales and Hancock, 2018). Highbush blueberry cultivars are further separated into northern and southern types, depending on their chilling requirements and winter hardiness (Hancock et al., 2008). Blueberries are now grown across the world, from North America to South America, Europe, China, Australia, and New Zealand. Their production has been increasing at a fast pace in the last decade, mainly because of a wider awareness among consumers about the positive effects of their consumption on human health (Coriolis, 2020). In the scientific literature there have been numerous reports supporting the anti-oxidant, anti-inflammatory, anti-proliferative, anti-obesity, and neuroprotective actions of blueberries (Norberto et al., 2013). These health-beneficial properties are mainly attributed to blueberries’ high content in flavonoids, and in particular anthocyanins (Prior et al., 1998; Grace et al., 2019; Kalt et al., 2020), which are also responsible for the red and blue pigmentation of mature fruits (Zifkin et al., 2012; Günther et al., 2020). Anthocyanins are composite molecules consisting of an anthocyanidin (or aglycone), linked with a sugar moiety. Common to known blueberry species are cyanidin, peonidin, delphinidin, malvidin, and petunidin-derived anthocyanins, which are conjugated with glucosides, galactosides, and arabinosides. In some cases, additional anthocyanins are reported. Some highbush varieties, in particular, produce anthocyanins linked with acylated glycosides (Yousef et al., 2014; Günther et al., 2020; Mengist et al., 2020). The accumulation of anthocyanins increases rapidly during fruit ripening and is controlled at the transcriptional level by members of the R2R3 MYB transcription factor family (Plunkett et al., 2018; Lafferty et al., 2022).

Species of the genus *Vaccinium* present a range of ploidy levels, from diploid (2n = 2x = 24), such as bilberries (*V. myrtillus* L.) and cranberries (*V. macrocarpon* Ait.), to tetraploid (2n = 4x = 48), such as most highbush and lowbush blueberries, and hexaploid (2n = 6x = 72), such as the rabbiteye blueberries (Hummer et al., 2015). Although there is still uncertainty about the origin of polyploidy in highbush blueberry (Colle et al., 2019), a number of studies suggested that polysomic inheritance is prevalent in this species (Draper and Scott, 1971; Krebs and Hancock, 1989; Lyrene et al., 2003). While there is an abundance of genetic mapping software available for diploids that can be used for allopolyploids, new tools have only recently been released for the construction of linkage maps and for quantitative trait locus (QTL) mapping using allele dosage information in polysomic polyploids (Grandke et al., 2017; Hackett et al., 2017; Bourke et al., 2018, 2021; Behrouzi and Wit, 2019; Mollinari and Garcia, 2019; da Silva Pereira et al., 2020; Amadeu et al., 2021). These new software tools have allowed the discovery of loci linked to complex traits in a number of important autopolyploid crops, such as forage grasses (Deo et al., 2020), rose (Cheng et al., 2021; Yu et al., 2021), potato (da Silva Pereira et al., 2021), sweet potato (da Silva Pereira et al., 2020; Gemenet et al., 2020; Oloka et al., 2021), kiwifruit (Tahir et al., 2020), and blueberry (Cappai et al., 2020; Mengist et al., 2021). Additionally, the release of two reference genomes for *V. corymbosum* (Gupta et al., 2015; Colle et al., 2019; Edger et al., 2022; Mengist et al., 2022) has facilitated the design of high-quality genotyping tools in this species.

Previous studies have reported QTLs for total anthocyanin concentration in a diploid cranberry population (Diaz-Garcia et al., 2018); however, the genetic determinism of anthocyanin content in blueberry is yet to be understood. In this work, we profiled the fruit anthocyanin diversity and content in a F_1_ segregating population of tetraploid highbush blueberry, and we identified associated QTLs and candidate genes. We used high-quality genotyping to build a high-density linkage map, and we implemented two of the newest R packages developed for genetic mapping in autopolyploids: polymapR and polyqtlR (Bourke et al., 2018, 2021).

## Materials and methods

### Mapping population, DNA extraction and genotyping

The mapping population used in this study was a bi-parental reciprocal cross made at The New Zealand Institute for Plant and Food Research Limited (PFR) in 2006 between the *V. corymbosum* cultivar “Nui” and the *V. corymbosum* open pollinated (OP) cultivar “Hortblue Petite.” A total of 360 offspring and the parents were grown in the research orchard at PFR, Motueka (41.0765°S, 172.9973°E) over three rows. Leaves were sampled from the parents (two replicates each) and 278 offspring, and DNA was extracted using the Kobayashi one leaf method (Kobayashi, 1998). The parents and the offspring were genotyped with the Capture-seq technology at RAPiD Genomics (Gainesville, FL, United States), using 31,630 blueberry 120-mer probes designed on the “W8520” reference genome (Benevenuto et al., 2019). Two technical replicates for each parent were genotyped. Parent DNAs were also whole genome sequenced using 10X Chromium by the Australian Genome Research Facility (AGRF, Brisbane, Australia). Short read sequence data from both the 10X Chromium and the Capture-seq were quality trimmed using BBduk in BBMAP v38.33 (Bushnell, 2014) and aligned to the “W8520” v1.3 genome (Mengist et al., 2022) using BWA-MEM in BWA v0.7.17 (Li, 2013) and samtools v1.9 (Danecek et al., 2021). Variant discovery was carried out using FreeBayes v1.3.1 (Garrison and Marth, 2012) (settings “-p 4 -C 5 -k –min-mapping-quality 10”) on both sets of data. Single nucleotide polymorphisms (SNPs) that matched between the two datasets (parental 10X Chromium and Capture-seq) were then selected and filtered for missing data at a 10% threshold.

### Linkage map construction

Quality filtering of the SNP data to prepare them for mapping and linkage map construction were performed in the R package polymapR v1.1.2 (Bourke et al., 2018), as described below. Unrelated offspring were identified visually with a principal component analysis (PCA) and were removed from the dataset, as well as the duplicated individuals (Pearson’s correlation coefficient _>_ 0.85); duplicated, monomorphic and distorted SNP markers were also discarded. Finally, only markers that had no missing calls in both parents and < 5% missing rate in the progeny were used for map construction. Homologue (H) and linkage group (LG) clusters were identified for each parent using the simplex × nulliplex (1 × 0) and the duplex × nullliplex (2 × 0) markers, respectively. Different combinations of LOD scores for H and LG clustering were tested and evaluated, until the best grouping was found; for some LGs, manual adjustments to the H clustering were performed in order to reach the desired combination of 12 LGs with 4 Hs each (corresponding to the 12 tetraploid chromosomes of blueberry). Afterward, all other types of markers were assigned to a LG and a H. Finally, pairwise recombination frequencies were estimated to find the order and the distance between the markers on each LG, and both a parental consensus map and a phased map were generated. At this step, a series of cleaning iterations were performed to remove markers with high nearest neighbor fit (nnfit) values, by visual inspection of position vs. nnfit plots. The quality of the genetic map was evaluated visually by generating diagnostic plots in polymapR, as well as Marey plots designed in Excel.

### Phenotyping

Blueberry fruits were harvested eating-ripe at Stage 8 (Zifkin et al., 2012) from 132 offspring and both parents, in summer 2019 and 2020 (128 offspring were harvested in both years). A minimum of 20 g per sample were then stored at −20°C until ready for extraction. In the 2020 season, additional parameters were recorded for 128 offspring. These included percentage of fully ripe berries on the bush at time of harvest (bush maturity), fruit diameter (calculated as average diameter of 10 berries) and fruit weight (calculated from the weight of 50 berries). In the laboratory, frozen whole fruit tissue was extracted into 100 mL of extraction solvent (ethanol, milli Q H_2_O, formic acid, 80/20/1, v/v/v). Approximately 20 g of fruit was extracted per genotype. Aliquots were diluted 10x with 1% formic acid/methanol prior to analysis via high-performance liquid chromatography-mass spectrometry (HPLC-MS) to identify anthocyanin compounds using the same method and conditions described in Günther et al. (2020). Total concentrations of anthocyanins grouped by their aglycone or by their glycoside were calculated for each sample, as well as the total concentration of all anthocyanins detected.

Phenotypic data evaluation and statistical analysis were performed in the R environment, using the packages corrplot v0.84 (Wei and Simko, 2021), ggplot2 v3.3.5 (Wickham, 2016), and rms v5.1.4 (Harrell, 2019). The 2020 anthocyanin concentration values were adjusted to account for the harvest data recorded. Ordinary least squares (OLS) models were fitted for each anthocyanin trait using scaled and centered covariates, including all 2 way linear interactions and non-linear terms for each covariate (linear tail-restricted cubic splines with 3 knots). The models were then checked for overfitting and updated, if necessary. Residuals from the updated models were then extracted and used as corrected phenotypes for 2020 for all following analyses, unless specified otherwise. Correlations among traits (both single compounds and totals, as well as harvest data from 2020) for each year and between years for each trait were evaluated using Spearman coefficient (raw data from both years were used for correlations). A PCA was run for each year using the values of the single anthocyanin compounds, and their contributions to the PC1 and PC2 were plotted. The distribution of all traits was visualized with density plots. The data from the 2 years were also combined and linear models with genotype and year as fixed effects were fitted for each phenotypic trait. Estimated marginal means were calculated for each genotype to average over the year effect using the R package emmeans v1.7.0 (Lenth, 2021).

### Quantitative trait loci mapping

The raw anthocyanin concentrations from 2019, the corrected phenotypes from 2020 and the estimated marginal means from the two seasons combined were used to perform QTL mapping with the R package polyqtlR v0.0.5 (Bourke et al., 2021), which was developed to directly use the phased genetic maps output of polymapR. First, the parental consensus map was thinned down to bins of 1 cM, selecting markers in each bin to maximize their distribution across the genome and across parental homologues. Then, identity-by-descent (IBD) probabilities between offspring were estimated at all marker positions and interpolated at a regular grid of positions with 1 cM spacing. QTL scan was performed through linear regression on IBD probabilities and LOD scores were calculated. Genome-wide significance thresholds were established through a permutation test with 1,000 permutations (α =0.05). Subsequent rounds of QTL scan were performed using the identified QTL peaks as co-factors. When no additional QTL was detected, the phenotypic variance explained (PVE) by the QTLs was calculated. Homologue-specific effects around QTL peaks were also visualized, and Bayesian Information Criterion (BIC) was used to compare QTL models. Finally, LOD curves for all QTLs identified and all traits analyzed were plotted on the genetic map using ggplot2.

### Candidate genes

Bed format was used to specify the chromosomic regions underlying the major QTLs, with confidence intervals defined as a 2 LOD drop from either side of the QTL peak. The function “bedtools intersect” from BEDTools v2.30.0 (Quinlan and Hall, 2010) was used to extract predicted gene structures within the QTL region, and gene coding sequences were extracted from the reference genome “W8520” v1.3 using GffRead v0.9.6 (Pertea and Pertea, 2020). Genes were then annotated using Mercator v3.6^1^ with keywords “acyltransferase,” “transferase,” “secondary metabolism.flavonoids,” “RNA biosynthesis.transcriptional regulation.MYB transcription factor superfamily.transcription factor (GARP),” “Dihydroflavonol-4-reductase (DFR),” or “secondary metabolism.phenylpropanoids.lignin biosynthesis.”

## Results

### Single nucleotide polymorphism detection and high-density linkage map construction

**FIGURE 1:**
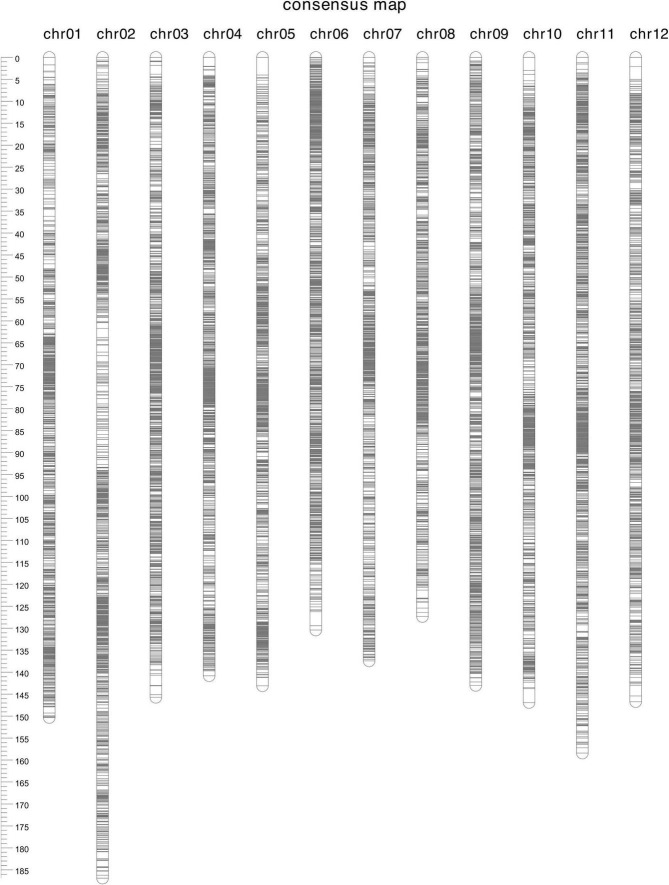
Linkage map of “Hortblue Petite” × “Nui”.

A total of 3,035,417 variants were discovered in a blueberry F_1_ population from a dataset comprising 10X Chromium sequences of the parents and Capture-seq reads of both parents and 278 offspring. After quality filtering and intersecting of the calls between the two datasets, 50,904 SNPs remained. In the polymapR, 29,526 distorted markers were identified and removed, as well as four offspring appearing as unrelated (Supplementary Figure 1A). Eight duplicate pairs of individuals were merged into a consensus genotype (designating the conflicting calls as missing) (Supplementary Figure 1B), leaving 266 unique genotypes to build the linkage map; the two replicates of the parents “Nui” and “Hortblue Petite” were also merged. A total of 19,332 polymorphic SNPs with nine different simplified segregation classes (Supplementary Figure 1C) passed all final marker filters. LOD scores of 15 and 19 were used, respectively, to create H and LG clusters in “Hortblue Petite,” and LOD scores of 15 and 12 were used in “Nui” for the respective clustering. Overall, 11,863 and 10,320 markers were assigned to one H and one LG in “Hortblue Petite” and “Nui,” respectively. A total of nine cleaning iterations were performed when building the consensus linkage map, which finally included 18,195 SNPs (18,033 after removing the co-mapping) and spanned 12 LGs over 1,756.0 cM (Table 1 and Figure 1). The LG numbers were assigned based on their correspondence with the 12 major scaffolds in the blueberry “W8520” v1.3 reference genome. The longest LG was LG2 (186.9 cM), and the shortest LG8 (127.3 cM). The average distance between markers was 0.1 cM, and the longest gap was 4.0 cM on LG5. Phasing was successful, generating eight homologue maps for each LG (H1, H2, H3, and H4 for “Hortblue Petite” and H5, H6 H7, and H8 for “Nui”; Supplementary Figure 2). According to the diagnostic plots produced by polymapR, the map appeared of high quality (i.e., with no markers showing high stress or appearing as outlier compared to neighboring ones), with the exception of LG2, whose central region (between 50 and 100 cM) showed some mis-mapping that could not be rectified with further cleaning (Supplementary Figure 3). Marey plots also showed high collinearity between the genetic and physical map (Supplementary Figure 4), with the exception of LG2, which had a region of 20 Mb at the end of the chromosome (between 46 and 66 Mb) that was inverted and mis-placed at the beginning of the genetic map (from 0 to 69.8 cM). Additionally, several SNPs mapping between 60.0 and 88.5 cM on LG2 had no collinearity with the physical map. The Marey plots also identified putative centromeric regions, which are characterized by low recombination rate and are then expressed as a plateau in the curve. For all LGs, the centromere appeared to be located in the center, except for LG6 where it was at the proximal end of the chromosome.

**TABLE 1:** Details of the linkage map for “Hortblue Petite” × “Nui”.

LG	Length (cM)	H1 no. SNPs	H2 no. SNPs	H3 no. SNPs	H4 no. SNPs	H5 no. SNPs	H6 no. SNPs	H7 no. SNPs	H8 no. SNPs	Unique no. SNPs
1	150.3	174	236	133	191	255	213	202	243	1,342
2	186.9	304	314	319	256	279	211	223	115	1,733
3	145.7	249	246	145	306	184	211	291	160	1,594
4	140.8	279	277	256	64	204	267	117	48	1,496
5	143.1	225	203	176	251	234	202	216	194	1,438
6	130.4	297	302	329	255	210	205	238	229	1,778
7	137.4	259	153	171	208	214	171	71	193	1,277
8	127.3	144	66	245	251	157	209	107	88	1,245
9	143.0	287	289	229	175	286	260	289	216	1,667
10	146.9	322	213	174	245	262	229	193	108	1,499
11	158.4	406	272	246	319	192	250	278	226	1,900
12	146.7	81	201	197	127	216	186	195	221	1,226
Total	1,756.9									18,195

The length and number (No.) of markers for each linkage group (LG), as well as the number of markers per homologue, are reported. H1, H2, H3, H4: homologues of “Hortblue Petite”; H5, H6, H7, H8: homologues of “Nui.” SNPs, single nucleotide polymorphism markers.

### Multiple anthocyanin compounds detected and quantified

**FIGURE 2:**
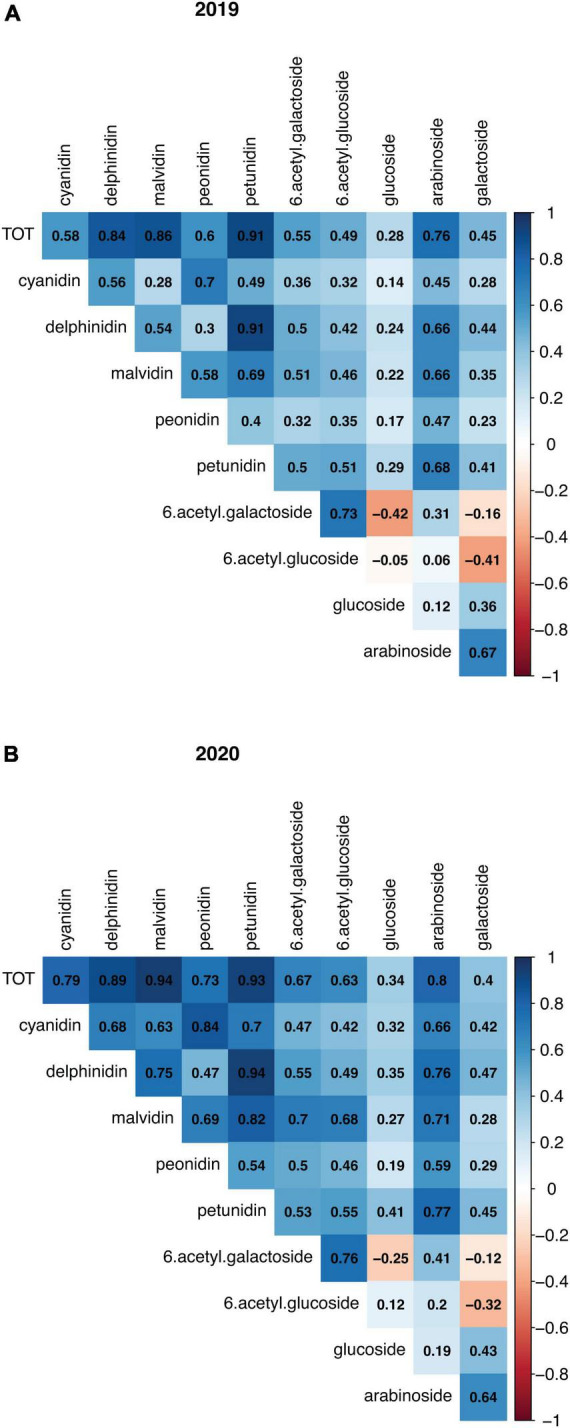
Correlation plots among anthocyanin compounds measured in “Hortblue Petite” × “Nui” population. The total concentrations for 10 groups of anthocyanin, as well as overall anthocyanin (TOT), in 2019 **(A)** and 2020 **(B)** were used for pairwise correlations.

**FIGURE 3:**
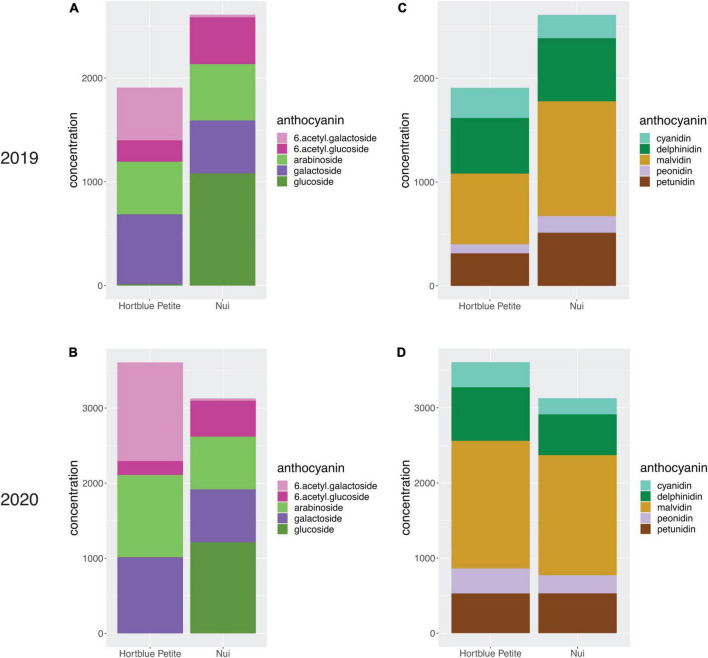
Comparison of anthocyanin content (μg/g) between “Hortblue Petite” and “Nui”. The overall anthocyanin content, as well as the proportions of each group of anthocyanin based on their sugar **(A,B)** and aglycone **(C,D)** moieties, in 2019 and 2020 are shown with stacked barplots.

A total of 25 anthocyanin compounds were identified and quantified in both years. These were composed of either a cyanidin, delphinidin, malvidin, peonidin, or petunidin aglycone conjugated via the C3 hydroxyl group to one of the following sugars: galactose, glucose, arabinose, and the two acylated forms of galactose and glucose. According to their anthocyanidin or sugar moiety, anthocyanins were combined into 10 groups and total concentrations were then calculated, as well as the overall total. When looking at the four harvest data types recorded in the year 2020, fruit diameter and fruit weight were significantly negatively correlated with anthocyanin concentrations, while correlations with harvest date were positive, and correlations with bush maturity were not significant (Supplementary Figure 5A). Fruit diameter and fruit weight were also strongly correlated with each other (*r*^2^ = 0.9) (Supplementary Figure 5B). Most single compounds (Supplementary Figure 6) and totals (Figure 2) were significantly (_ρ<0.05_) positively correlated, with a few exceptions. Notably, anthocyanins containing (6-acetyl)galactose were significantly negatively correlated with those containing the non-acylated glucose, and *vice versa* those with (6-acetyl)glucose were negatively correlated with anthocyanins containing non-acylated galactose. For all traits, concentrations between the 2 years were positively correlated (Supplementary Figure 7). When running a PCA using the concentrations of the single anthocyanin compounds, large variation appeared to be accounted for by the PC1 and PC2 (31.3 and 22.3%, respectively, in 2019, and 33.5 and 28.9% in 2020). In both PCA plots, it was possible to identify two main clusters of samples along the PC1, and the main contribution derived from anthocyanins containing (6-acetyl)galactose and (6-acetyl)glucose sugars (Supplementary Figure 8). Contribution to the PC2 was mainly given by compounds with the sugar moieties galactose and arabinose, although a clear distinction was not evident in the 2020 data. Anthocyanin concentrations for the two parents were variable across the 2 years; however, compounds with acylated sugars accounted for approximately one third of anthocyanins in “Hortblue Petite” in both years, while they were proportionally less abundant in “Nui,” which had instead a higher content of glucosides than “Hortblue Petite” (Figures 3A,B). The proportion of aglycones was similar between the two parents and across both years (Figures 3C,D). Transgressive lines, i.e., offspring with higher and lower values than the parents, were present (Supplementary Table 1). Approximately 60% of the offspring and the parent “Nui” comprised all 25 anthocyanins, while the remaining individuals and the parent “Hortblue Petite” comprised only 12–24 compounds, although with some inconsistencies across the 2 years. Density plots showed that many of the traits were normally distributed, except for the anthocyanins containing acylated sugar moieties, which predominantly had distributions close to bimodality (Supplementary Figure 9).

### Two major quantitative trait loci identified for anthocyanin content

**FIGURE 4:**
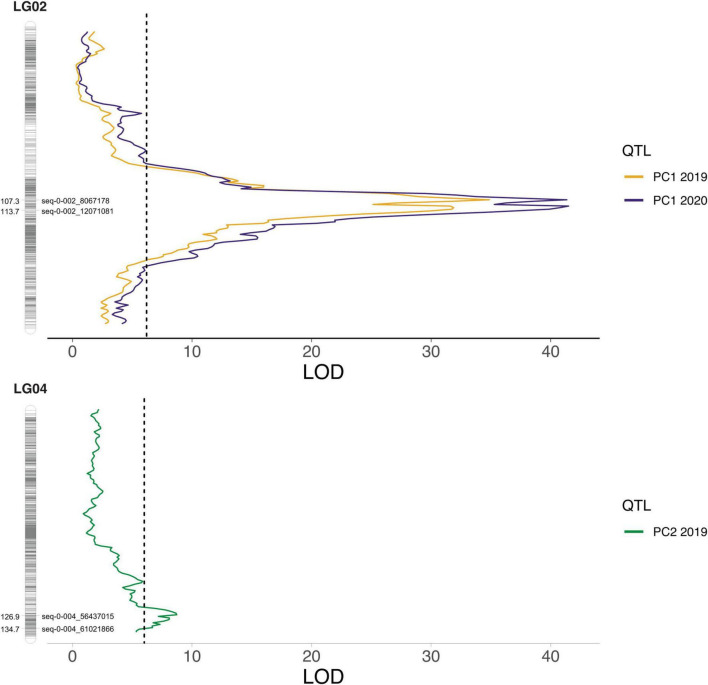
Quantitative trait locus (QTL) peaks for Principal Component (PC) 1 and PC2 in 2019 and 2020. On both linkage groups (LGs) the markers delimiting the –2 LOD confidence interval are highlighted.

The raw (non-adjusted) anthocyanin concentrations from 2019, the corrected phenotypes from 2020, as well as the PC1 and PC2 values from both years, and the estimated marginal means from the two seasons combined were used for QTL mapping in polyqtlR. Two major QTLs were consistently discovered with the 2019 and 2020 data, as well as with the overall means, on LGs 2 and 4 (Figure 4, Supplementary Figure 10, and Supplementary Table 2). Genome-wide significance thresholds ranged between 5.5 and 6.3 LOD, and the QTL peaks were between 6.2 and 41.5 LOD on LG2 and between 6.0 and 14.2 LOD on LG4. The PVE was estimated between 9.0 and 77.5% for LG2, and between 4.2 and 39.0% for LG4. The LG2 QTL peaks mapped between 102 and 113 cM (11 cM window) in 2019, 104, and 119 cM (15 cM window) in 2020, and 107 and 116 cM (9 cM window) in the 2-year combined data. Notably, consistently identified peaks for the LG2 QTLs shifted of no more than 6 cM from one year to the next, with the exception of QTLs for petunidin 3-galactoside, which had a shift of 10 cM between 2019 and 2020. For the QTLs mapped to LG4, the peaks were recorded between 120 and 136 cM (16 cM window) in 2019, between 123 and 136 cM (13 cM window) in 2020, and between 124 and 138 cM (14 cM window) in the 2-year combined data. The LG4 QTL peak positions were also consistent across datasets, with shifts of no more than 9 cM between 2019 and 2020. In all three datasets, many of the traits were associated with both LG2 and LG4 QTLs (Supplementary Figure 10). Minor QTLs were also detected on LGs 1, 7, 8, and 12 in 2019, LGs 3, 7, 8, 9, and 12 in 2020, and LGs 7, 8, 9, and 12 in the 2-year combined data. Of these, the only QTLs that were consistently detected in all three datasets were on LG7 for malvidin 3-(6-acetyl)glucoside and total (6-acetyl)glucosides, with peaks at 123 cM, and on LG12 for petunidin 3-(6-acetyl)glucoside, with peaks between 104 and 106 cM. PVEs calculated with the all-QTLs model for trait associated with more than one QTL ranged between 33.9 and 74.4%.

**FIGURE 5:**
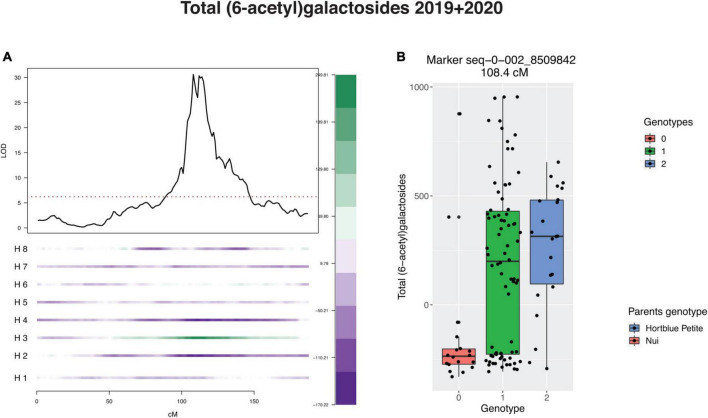
Effect of major quantitative trait locus (QTL) on linkage group 2 (LG2) for total (6-acetyl)galactosides. **(A)** Negative (purple) and positive (green) effect of each homologue (H). H1, H2, H3, H4: homologues of “Hortblue Petite”; H5, H6, H7, H8: homologues of “Nui.” **(B)** Boxplot of phenotype grouped by genotype at the single nucleotide polymorphism (SNP) marker at the peak of the QTL.

The LG2 QTL was linked to the homologue numbered as H3 of the parent “Hortblue Petite,” which had a positive effect on the concentration of all anthocyanins containing acylated sugars (Figure 5 and Supplementary Table 3). The same allele had instead a negative effect on the anthocyanins containing non-acylated forms of glucoside and galactoside. For most of the traits this allele was also estimated to have additive, rather than dominant, action. Analysis of the effect of the LG4 QTLs was less clear, with contributions detected from a number of different homologues from both parents (Supplementary Table 3).

### Candidate genes in confidence interval of major quantitative trait loci

Candidate genes were identified in the two major QTL regions (Supplementary Table 4). LG2 QTL spanned a region between 8 and 11 Mbp of chromosome 2 of the “W8520” v1.3 reference genome (Mengist et al., 2022), which contains 134 genes and 151 mRNA. Four genes were annotated with functions of interest, in particular: two acetyltransferase genes, one MYB transcription factor (GARP subfamily), and one dihydroflavonol 4-reductase (DFR) gene. LG4 QTL covered the region between 57 and 61 Mbp on chromosome 4 and enclosed 181 genes and 211 mRNAs. Among them, 31 genes were detected with relevant functions: four acetyltransferase genes, one MYB (MYB36), two DFR, three secondary metabolism (two for flavonol-3-O-rhamnosyltransferase and one for p-coumaroyl-CoA biosynthesis), and 21 genes annotated as other types of transferase.

## Discussion

To the best of our knowledge, this is the most comprehensive study of the genetic determinism of anthocyanin content in highbush blueberry. The construction of a high-density and high-quality linkage map, made possible by the recent release of tools for genetic mapping in polyploids, and an in-depth chemical analysis of blueberry fruits in a large segregating population, have allowed the discovery of QTLs associated with anthocyanin content and the underlying candidate genes.

### High-density and high-quality linkage map for tetraploid blueberry

The linkage map built in this study is one of the few high-density genetic maps for tetraploid blueberry to be published. The first was reported by McCallum et al. (2016), who built two parental maps for a F_1_ segregating population between a northern highbush and a southern highbush cultivar using simple sequence repeat (SSR) and SNP markers. This work was carried out prior to the release of tools able to utilize allele dosages in the construction of linkage maps, therefore only a portion of the genetic information could be used. The two maps included 785 and 536 markers, respectively, and spanned 12 LGs over 1,621 cM and 20 LGs over 1,610 cM. More recently, Cappai et al. (2020) and Mengist et al. (2021) reported the construction of linkage maps for tetraploid highbush blueberry populations, which included 11,292 SNP markers over 1,953.97 cM and 17,438 SNP over 1,397 cM, respectively. Both studies made use of Capture-seq, demonstrating the reliability of this technology for high-quality genotyping in autopolyploid species. Additionally, they applied recently released software for genetic map construction, specifically mappoly and polymapR. The genetic map presented in this study, with 18,195 SNPs over 1,756.0 cM (Table 1 and Figure 1), is therefore in line with the one reported by Mengist et al. (2021).

When looking at the Marey plots, the genetic map showed overall good collinearity with the diploid blueberry “W8520” reference genome, except for a mismatch on a large region at the top of LG2, from 0 to 69.8 cM (Supplementary Figure 4), which is also likely the cause of the higher length of this LG than the others. Both Cappai et al. (2020) and Mengist et al. (2021) described mismatches between the genetic and the physical map at the telomere in LG2; however, these were only minor rearrangements. In contrast, here it appears that a 20 Mb-long chromosome segment had incurred both a translocation and an inversion. All four parental homologues and all marker segregation classes showed the same pattern. Additionally, the map diagnostic plots highlighted issues on LG2 (Supplementary Figure 3), specifically in a region between 60.0 and 86.6 cM where the marker density was also low (Figure 1 and Supplementary Figure 2) and there was no collinearity with the physical map (Supplementary Figure 4). This region corresponds to the break point of the rearranged segment. While a chromosomal re-arrangement between the parents of the mapping population and the reference genome is possible, the diagnostic plots suggest that errors at the marker ordering step of the map construction are a more likely explanation in this case. These can be caused by genotyping errors or dosage scoring errors of the SNP markers in this region. Minor re-arrangements were also observed at the telomere of LG5, as also reported by Mengist et al. (2021), and around the centromere in LG8. Additionally, in LG6 the low recombination region, typical of the centromere, was located at the beginning of the LG, and not in the middle as expected. A similar pattern was observed for LG6 by Mengist et al. (2021). Other chromosomal re-arrangements reported by Cappai et al. (2020) and Mengist et al. (2021) were not observed here. It is important to note, however, that these authors compared their maps to the tetraploid reference genome “Draper” (Colle et al., 2019), while that of the diploid “W8520” was used in this study.

### Detailed anthocyanin profile of a highbush mapping population

In this study, 25 different anthocyanins were identified and quantified in the fruits of a highbush blueberry mapping population over two seasons. Fewer compounds were identified by Mengist et al. (2020), who analyzed 98 *V. corymbosum* accessions, other than other *Vaccinium* species, and did not find any trace of cyanidin 3-(6-acetyl)galactoside, delphinidin 3-(6-acetyl)galactoside, peonidin 3-arabinoside, petunidin 3-(6-acetyl)galactoside, and petunidin 3-(6-acetyl)glucoside. This is likely due to the different analytical methodologies used, as Mengist et al. (2020) used HPLC-photodiode array detection (HPLC-PDA) analysis, while HPLC-MS, which is more sensitive and does not necessitate complete chromatographic separation for compound identification, was used in this study. The variation observed for total anthocyanins across the tetraploid accessions studied by these authors (ninefold) was higher than that measured in the “Hortblue Petite” × “Nui” progeny (sixfold in 2019 and sixfold in 2020), consistent with the wider genetic background of the genotypes screened by Mengist et al. (2020).

Environmental factors, and in particular light and temperature, are known to affect anthocyanin accumulation in a variety of crops (Åkerström et al., 2010; Lin-Wang et al., 2011; Karppinen et al., 2016; Henry-Kirk et al., 2018; Liu et al., 2019). However, in this study phenotypic data between the 2 years were highly correlated (Supplementary Figure 7). This is consistent with the moderate to high broad-sense heritability values reported by Mengist et al. (2020), which suggested the importance of genetic control on these traits despite the known influence of the environment. Identification in the present study of two major QTLs stable across the 2 years further supports this hypothesis.

### Effect of fruit size on anthocyanin content

Fruit size, here determined as fruit weight and diameter, was significantly negatively correlated with anthocyanin content (Supplementary Figure 5). This was expected, as anthocyanins are known to be localized in the skin of blueberries (Zifkin et al., 2012; Scalzo et al., 2015; Plunkett et al., 2018; Günther et al., 2020), and similar correlations were observed in previous studies (Yousef et al., 2014; Gündüz et al., 2015; Mengist et al., 2020). Recording fruit weight and diameter in the second season of this study was important to evaluate if correcting anthocyanin content for these factors would affect the power and position of the QTLs. Overall, only small differences between 2019 and 2020 QTLs were observed (Supplementary Figure 10 and Supplementary Table 2). The two major QTLs on LG2 and LG4 were predominantly consistent across the 2 years, with similar LOD scores and PVE values, and peaks shifted of no more than 6 cM on LG2 for most traits, and just up to 9 cM on LG4. On the contrary, notable differences were observed for the minor QTLs, with the one on LG1 only significant in 2019, and those on LGs 3 and 9 only significant in 2020. QTLs on LG8 were detected in both years, although they mapped to different positions. However, minor QTLs on LGs 7 and 12 were consistent across the 2 years, and they were confirmed when using the means from the two seasons. It is difficult to infer if differences between the 2 years were because of the phenotypic correction performed in 2020 but not in 2019, or because of environmental differences. However, the major LG2 and LG4 QTLs, as well as the minor LG7 and LG12 QTLs, were stable across years and warrant further investigation.

### Two major quantitative trait loci control anthocyanin content and composition in blueberry

A large variation in concentrations was observed for each of the measured anthocyanin compounds in the “Hortblue Petite” _×_ “Nui” offspring, and the presence of transgressive lines confirmed the polygenic nature of these traits (Supplementary Table 1). The parents of the mapping population were not very dissimilar in terms of total anthocyanin concentration, and variability was observed across the two seasons (Figure 3). However, a consistent pattern could be observed by grouping the anthocyanins by their sugar moiety. Interestingly, “Hortblue Petite” had a higher concentration of (6-acetyl)galactosides than “Nui,” which instead had more (6-acetyl)glucosides, as well as more non-acylated glucosides. Overall, anthocyanins with acylated sugars were present in a larger proportion in “Hortblue Petite” than in “Nui.” Two major QTLs were detected on LG2 and LG4 (Figure 4), and they were consistent for a number of different anthocyanin compounds and were stable across the 2 years (Supplementary Figure 10). The strongest QTLs, both in terms of LOD score and PVE, were found on LG2 for the PC1 in both 2019 and 2020. Notably, the large variation represented by the PC1 was mainly driven by anthocyanins containing acylated sugar moieties (Supplementary Figure 8). Additionally, QTLs mapped to LG2 for compounds with acylated glucose or galactose had overall higher LOD and PVE values than for the other traits (Supplementary Table 2). Given these results, the two acetyltransferase genes identified within the LG2 QTL interval appear particularly interesting and follow-up studies should focus on validating their putative function on acylated anthocyanin accumulation. Concentrations of these types of anthocyanins also had the highest correlation coefficients between the 2 years (Supplementary Figure 7), and the QTLs were largely consistent over time. This might be because acylation improves the stability of the anthocyanin molecule to heat and light exposure (Giusti and Wrolstad, 2003), which may therefore decrease variability across different environments. The increased stability of acylated anthocyanins makes them also more desirable for the pigment and food industries (Giusti and Wrolstad, 2003; Jokioja et al., 2021). More variation was observed on the type of anthocyanins associated with the LG4 QTL, which overall was also smaller and had a lower effect than the one on LG2 (Figure 4 and Supplementary Table 3). Furthermore, many of the traits associated with the LG4 QTL were also associated with the LG2 QTL, and the two loci appeared to have epistatic effects, since the PVE for the all-QTLs model for these traits was different (in some cases higher, in other lower) to the PVE for the simple sum of the two single-QTL models (Supplementary Table 2).

A major QTL was previously mapped to blueberry LG2 for color (Qi et al., 2021), and anthocyanins are known to be linked to skin pigmentation in blueberry fruits (Zifkin et al., 2012; Plunkett et al., 2018). Blueberry chromosome 2 regions were also significantly associated with volatile emissions, in particular 2-nonanone, 2-undenonanone and eucalyptol (Ferrão et al., 2020), whose biosynthesis is linked to acetyl-CoA-benzylalcohol acetyltransferase (Dudareva et al., 1998). However, since the reference genome of “Draper” was used in these two studies, it is difficult to determine if those regions co-map with the one discovered in the present study. A major QTL for total anthocyanin content was also identified on chromosome 3 of cranberry (*V. macrocarpon*) (Diaz-Garcia et al., 2018, 2021), which corresponds to chromosome 8 of the blueberry and bilberry reference genomes (Wu et al., 2022). In the present study, minor QTLs were found in both 2019 and 2020 on LG8 for anthocyanins containing the galactose sugar moiety, although at inconsistent positions. Minor QTLs were also found on the cranberry LGs 6 and 11 (Diaz-Garcia et al., 2018), corresponding, respectively, to chromosomes 1 and 4 of blueberry, where a minor and a major QTL were detected in the present work.

It was not possible to calculate heritability in this study; however, previous works have estimated moderate to high (40–80%) broad-sense heritabilities for anthocyanin compounds in tetraploid blueberries (Mengist et al., 2020). The QTL on LG2 by itself accounted for up to 77.5% of phenotypic variance for single compounds, while PVEs for all-QTL models reached 74.4%. Although the specific heritability for this mapping population should be estimated in order to make better comparisons, these results suggest that a large portion of the genetic loci controlling anthocyanin content were identified in this study. For most of the traits, in both years the LG2 QTL effect was linked to H3 of the parent “Hortblue Petite” (Figure 5 and Supplementary Table 3). Additionally, the H3 allele contributed positively to all anthocyanin compounds containing (6-acetyl)galactose and (6-acetyl)glucose moieties, i.e., it led to an increase in these metabolite concentrations, while the opposite was observed for anthocyanins containing galactose and glucose sugar moieties (Supplementary Table 3).

## Conclusion

In this study, novel QTLs controlling anthocyanin content were identified in a highbush blueberry population. The major effect of the LG2 QTL is particularly encouraging for the design of markers to apply in marker-assisted selection practices. Furthermore, the fact that this QTL was shown to specifically control the concentration of compounds with acylated sugars is very promising for the development of cultivars with improved fruit quality characteristics.

## Data availability statement

The datasets presented in this study can be found in the Genome Database for Vaccinium (GDV) at the following link: https://www.vaccinium.org/bio_data/6147265.

## Author contributions

RE and DC: conceptualization. SM, CG, TM, RE, and DC: methodology. SM, ST, PM, and CD: formal analysis. SM, SC, MK, TF, and JT: investigation. JT: resources. SM: writing—original draft preparation and visualization. RE: funding acquisition. All authors: writing—review and editing.

## Footnotes

1
https://www.plabipd.de

## Supplementary material

Supplementary Figure 1 Individual and marker filtering in polymapR for ‘Hortblue Petite’ × ‘Nui’ population. **(A)** Principal component analysis of parents and progeny to identify unrelated offspring. **(B)** Plot of sample pairwise correlation coefficients to identify and merge duplicated individuals (threshold at 0.85). **(C)** Simplified segregation classes for the 19,332 single nucleotide polymorphism (SNP) markers that passed all filters. Simplified segregation classes: 1 × 0 includes 3 × 0, 3 × 4 and 1 × 4 markers; 2 × 0 includes 2 × 4 markers; 0 × 1 includes 0 × 3, 4 × 3 and 4 × 1 markers; 1 × 1 includes 3 × 3 markers; 2 × 1 includes 4 × 2 markers; 1 × 2 includes 3 × 2 markers; 1 × 3 includes 3 × 1 markers.

Supplementary Figure 2 Phased linkage map of ‘Hortblue Petite’ × ‘Nui’ population. For each of the 12 linkage groups (LGs), the map for the four ‘Hortblue Petite’ homologues are shown in purple, the map for the four ‘Nui’ homologues are shown in blue, and the consensus is shown in black.

Supplementary Figure 3 ‘Hortblue Petite’ × ‘Nui’ linkage map diagnostic plots produced by polymapR. For each one of the 12 linkage groups (LGs), three plots are shown: (i) the upper graph shows a plot of the difference between the pairwise estimates and the effective estimate of recombination frequency (δ_(*r*)_) versus the LOD score for each pairwise estimate; (ii) the bottom left plot shows the comparison between a marker’s map position and the recombination frequency estimates to all other markers; (iii) the bottom right plot shows the comparison between a marker’s map position and the LOD values to all other markers.

Supplementary Figure 4 Marey plots showing collinearity between the ‘Hortblue Petite’ × ‘Nui’ genetic and the physical map. Single nucleotide polymorphism (SNP) positions on the *Vaccinium corymbosum* ‘W8520’ v1.3 reference genome were used to indicate physical map.

Supplementary Figure 5 Correlation plots among phenotypic data collected in 2020 on ‘Hortblue Petite’ × ‘Nui’ population. **(A)** Pairwise correlations among total anthocyanin concentrations (μg/g), fruit diameter (DiamAvg), fruit weight (FrtWt.F), harvest date (HarvDateJul) and bush maturity (HarvPercentBlue). **(B)** Pairwise correlations among fruit weight, bush maturity, fruit diameter (avg Diameter) and harvest date. The upper panels show correlation coefficients; the diagonal panels show density plots; the lower panel show scatter plots.

Supplementary Figure 6 Correlation plots among single anthocyanin compounds in ‘Hortblue Petite’ × ‘Nui’ population. For both 2019 **(A)** and 2020 **(B)**, raw data (anthocyanin concentrations in μg/g) were used.

Supplementary Figure 7 Scatter plots showing correlation between two years of phenotyping for anthocyanin content in ‘Hortblue Petite’ × ‘Nui’ population. For each anthocyanin compound and totals, scatter plots between 2019 (x axis) and 2020 (y axis) raw data (anthocyanin concentrations in μg/g) are displayed. Spearman correlation coefficients are reported in blue. cy, cyanidin; del, delphinidin; mal, malvidin; peo, peonidin; pet, petunidin; ac, acetyl; gal, galactoside; glu, glucoside; ar, arabinoside; tot, total.

Supplementary Figure 8 Principal component analysis (PCA) of single anthocyanin concentrations (μg/g) from ‘Hortblue Petite’ × ‘Nui’ population in years 2019 and 2020. For each year, a PC1 versus PC2 plot is shown, as well as the variable contribution (%) to the first and second PC.

Supplementary Figure 9 Density plots for each trait in 2019 and 2020. For both years, raw data (anthocyanin concentrations in μg/g) were used. For 2020, also distribution of phenotypes adjusted for harvest data (residuals of fitted models) are shown.

Supplementary Figure 10 Comparison of quantitative trait loci (QTLs) across years and traits. For each anthocyanin trait, the LOD curves on all 12 linkage groups (LGs) are shown for 2019, 2020 and for the two-year combined data.

Supplementary Table 1 Phenotypic data collected in 2019 and 2020 in “Hortblue Petite” × “Nui” population. Anthocyanin concentrations are reported for the two parents, as well as minimum and maximum values for the offspring. Values for four harvest traits are also reported for 2020.

Supplementary Table 2 Details of quantitative trait loci (QTLs) identified in “Hortblue Petite” × “Nui” population for anthocyanin content. For year 2019, 2020 and for the adjusted means across the 2 years, the QTL position, LOD score and threshold, and the phenotypic variance explained (PVE) are reported for each trait.

Supplementary Table 3 Effects of major quantitative trait loci (QTLs). For year 2019, 2020 and for the adjusted means across the 2 years the homologue linked to the QTL and its estimated contribution and gene action are reported. H1, H2, H3, H4: homologues of “Hortblue Petite”; H5, H6, H7, H8: homologues of “Nui”.

Supplementary Table 4 Candidate genes identified in the interval of the two major quantitative trait loci (QTLs).
