# Supplementary material for: High-density linkage map construction in an autotetraploid blueberry population and detection of quantitative trait loci for anthocyanin content
Source: Front Plant Sci. 2022 Sep 23;13:965397. doi: 10.3389/fpls.2022.965397 (PMC9555082; doi:10.3389/fpls.2022.965397)
Supplement: Supplementary Figure 1 — Individual and marker filtering in polymapR for ‘Hortblue Petite’ × ‘Nui’ population. (A) Principal component analysis of parents and progeny to identify unrelated offspring. (B) Plot of sample pairwise correlation coefficients to identify and merge duplicated individuals (threshold at 0.85). (C) Simplified segregation classes for the 19,332 single nucleotide polymorphism (SNP) markers that passed all filters. Simplified segregation classes: 1 × 0 includes 3 × 0, 3 × 4 and 1 × 4 markers; 2 × 0 includes 2 × 4 markers; 0 × 1 includes 0 × 3, 4 × 3 and 4 × 1 markers; 1 × 1 includes 3 × 3 markers; 2 × 1 includes 4 × 2 markers; 1 × 2 includes 3 × 2 markers; 1 × 3 includes 3 × 1 markers. [file Data_Sheet_1.PDF]

Supplementary Figures

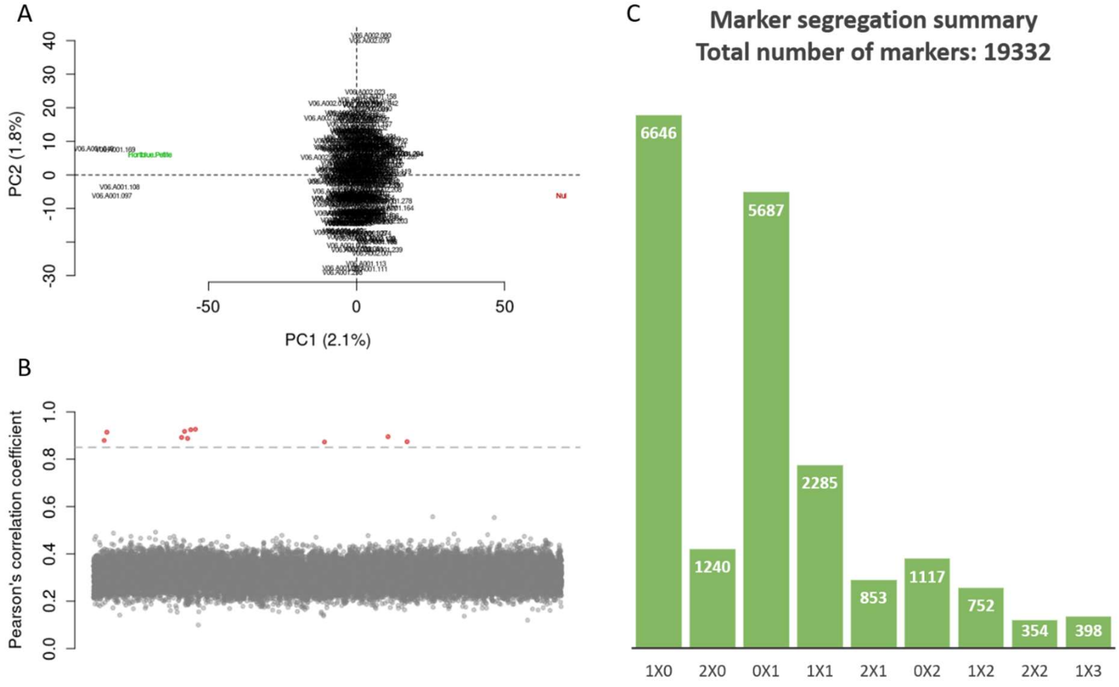

Supplementary Figure 1. Individual and marker filtering in polymapR for 'Hortblue Petite' × 'Nui' population. A) Principal Component Analysis of parents and progeny to identify unrelated offspring. B) Plot of sample pairwise correlation coefficients to identify and merge duplicated individuals (threshold at 0.85). C) Simplified segregation classes for the 19,332 single nucleotide polymorphisms (SNPs) that passed all filters. Simplified segregation classes: 1×0 includes 3×0, 3×4 and 1×4 markers; 2×0 includes 2×4 markers; 0×1 includes 0×3, 4×3 and 4×1 markers; 1×1 includes 3×3 markers; 2×1 includes 4×2 markers; 1×2 includes 3×2 markers; 1×3 includes 3×1 markers.

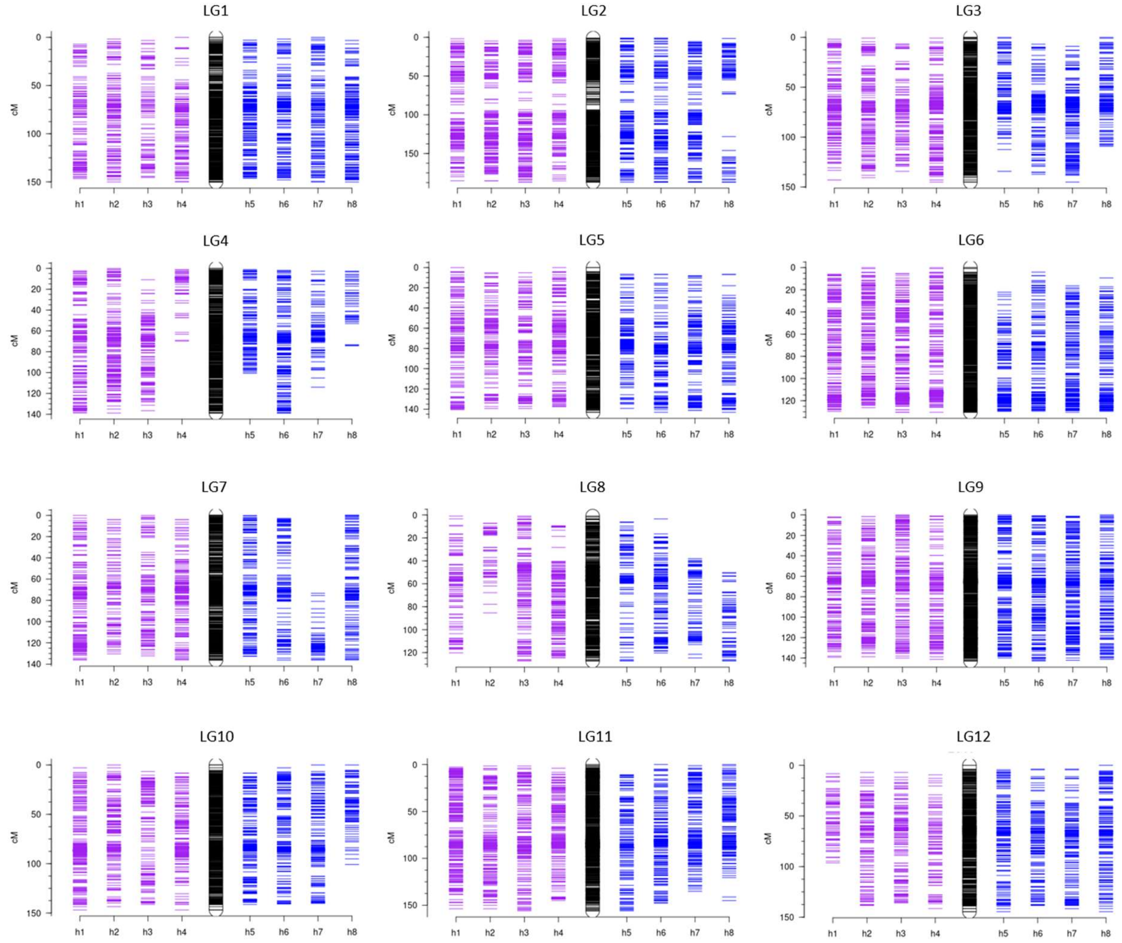

Supplementary Figure 2. Phased linkage map of 'Hortblue Petite' × 'Nui' population. For each of the 12 linkage groups (LGs), the map for the four 'Hortblue Petite' homologues are shown in purple, the map for the four 'Nui' homologues are shown in blue, and the consensus is shown in black.

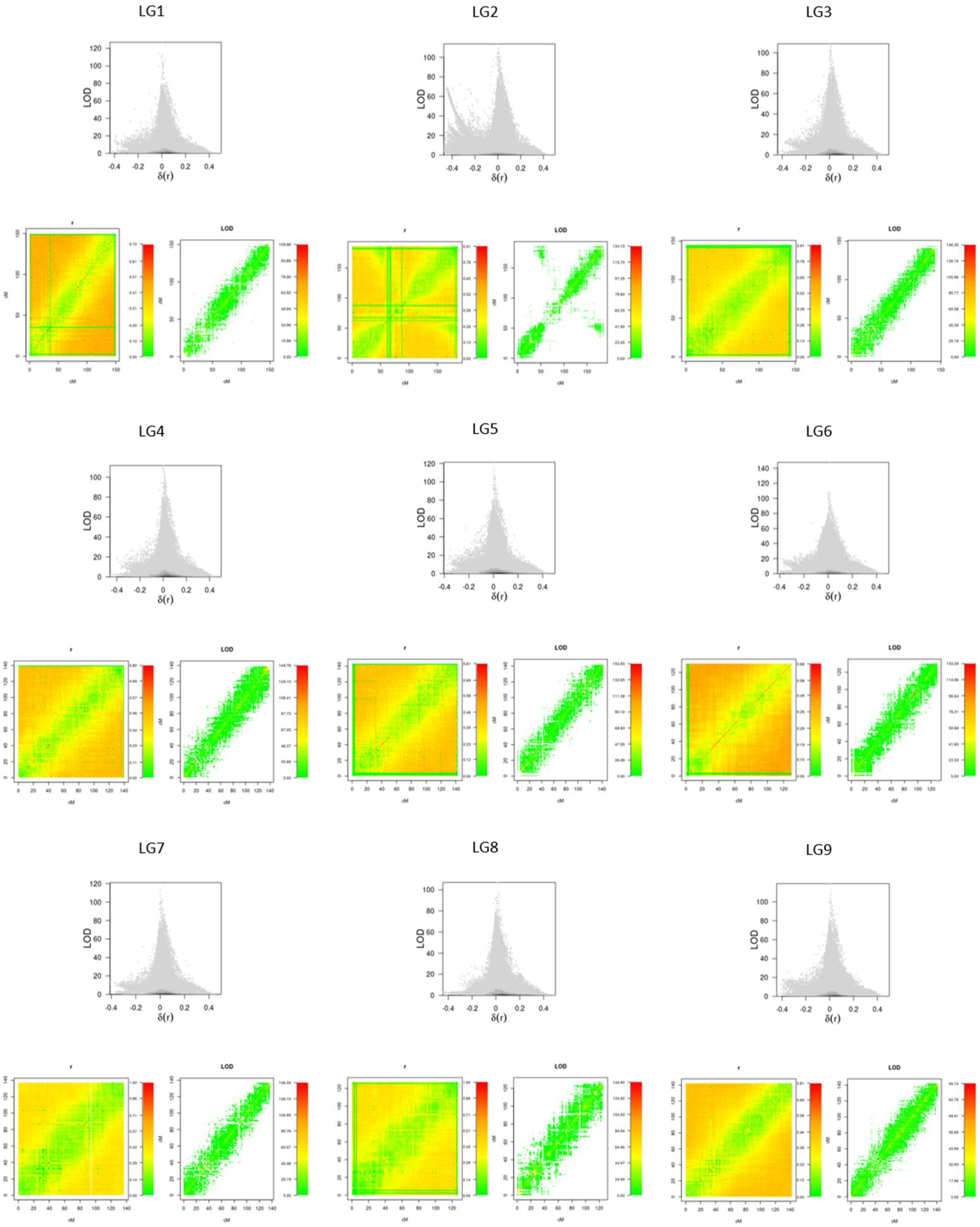
LG1
LG2
LG3
LG4
LG5
LG6
LG7
LG8
LG9
LOD
δ(r)
r
cM

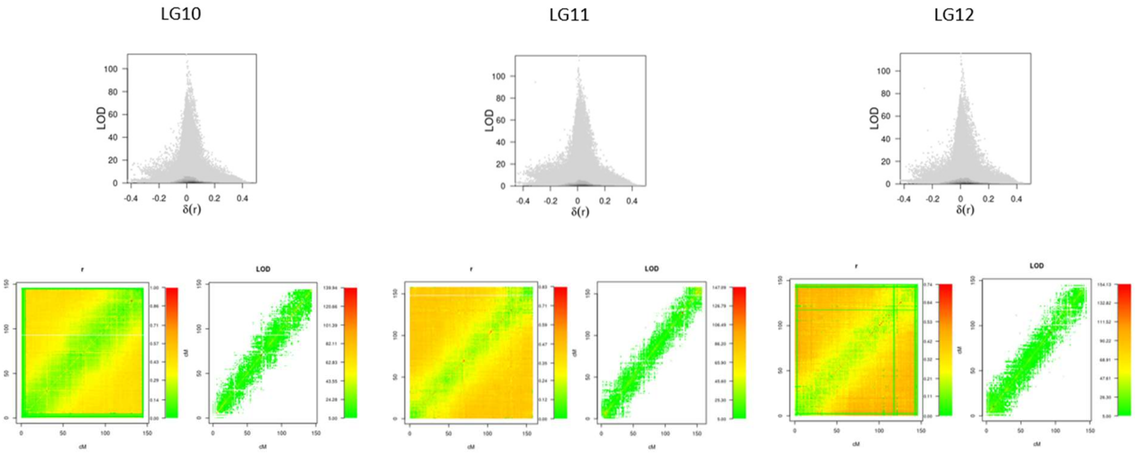

Supplementary Figure 3. 'Hortblue Petite' × 'Nui' linkage map diagnostic plots produced by polymapR. For each one of the 12 linkage groups (LGs), three plots are shown: i) the upper graph shows a plot of the difference between the pairwise estimates and the effective estimate of recombination frequency ($\delta_{(r)}$) versus the LOD score for each pairwise estimate; ii) the bottom left plot shows the comparison between a marker's map position and the recombination frequency estimates to all other markers; iii) the bottom right plot shows the comparison between a marker's map position and the LOD values to all other markers.

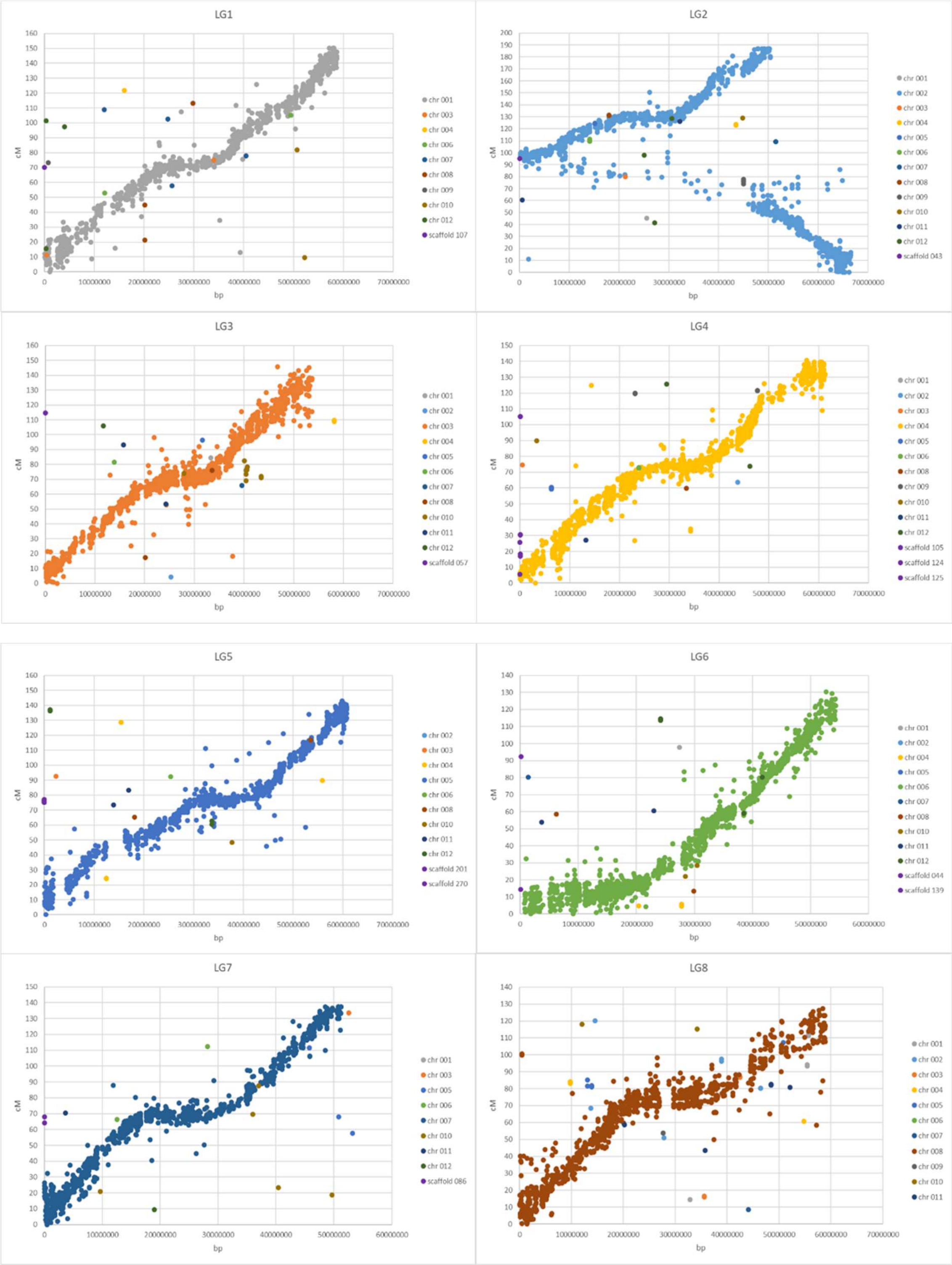

LG1
cM
bp
chr 001
chr 003
chr 004
chr 006
chr 007
chr 008
chr 009
chr 010
chr 012
scaffold 107
LG2
chr 001
chr 002
chr 003
chr 004
chr 005
chr 006
chr 007
chr 008
chr 009
chr 010
chr 011
chr 012
scaffold 043
LG3
chr 001
chr 002
chr 003
chr 004
chr 005
chr 006
chr 007
chr 008
chr 010
chr 011
chr 012
scaffold 057
LG4
chr 001
chr 002
chr 003
chr 004
chr 005
chr 006
chr 008
chr 009
chr 010
chr 011
chr 012
scaffold 105
scaffold 124
scaffold 125
LG5
chr 002
chr 003
chr 004
chr 005
chr 006
chr 008
chr 010
chr 011
chr 012
scaffold 201
scaffold 270
LG6
chr 001
chr 002
chr 004
chr 005
chr 006
chr 007
chr 008
chr 010
chr 011
chr 012
scaffold 044
scaffold 139
LG7
chr 001
chr 003
chr 005
chr 006
chr 007
chr 010
chr 011
chr 012
scaffold 086
LG8
chr 001
chr 002
chr 003
chr 004
chr 005
chr 006
chr 007
chr 008
chr 009
chr 010
chr 011

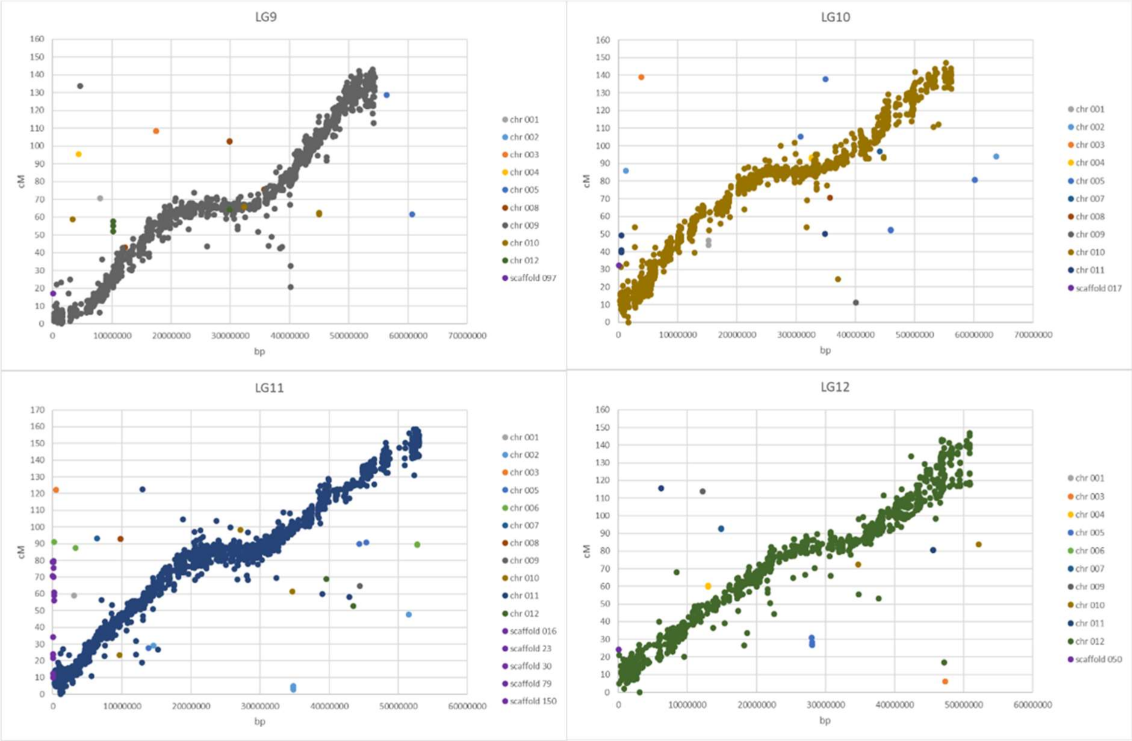

Supplementary Figure 4. Marey plots showing collinearity between the ‘Hortblue Petite’ × ‘Nui’ genetic and the physical map. Single nucleotide polymorphism (SNP) positions on the *Vaccinium corymbosum* ‘W8520’ v1.3 reference genome were used to indicate physical map.

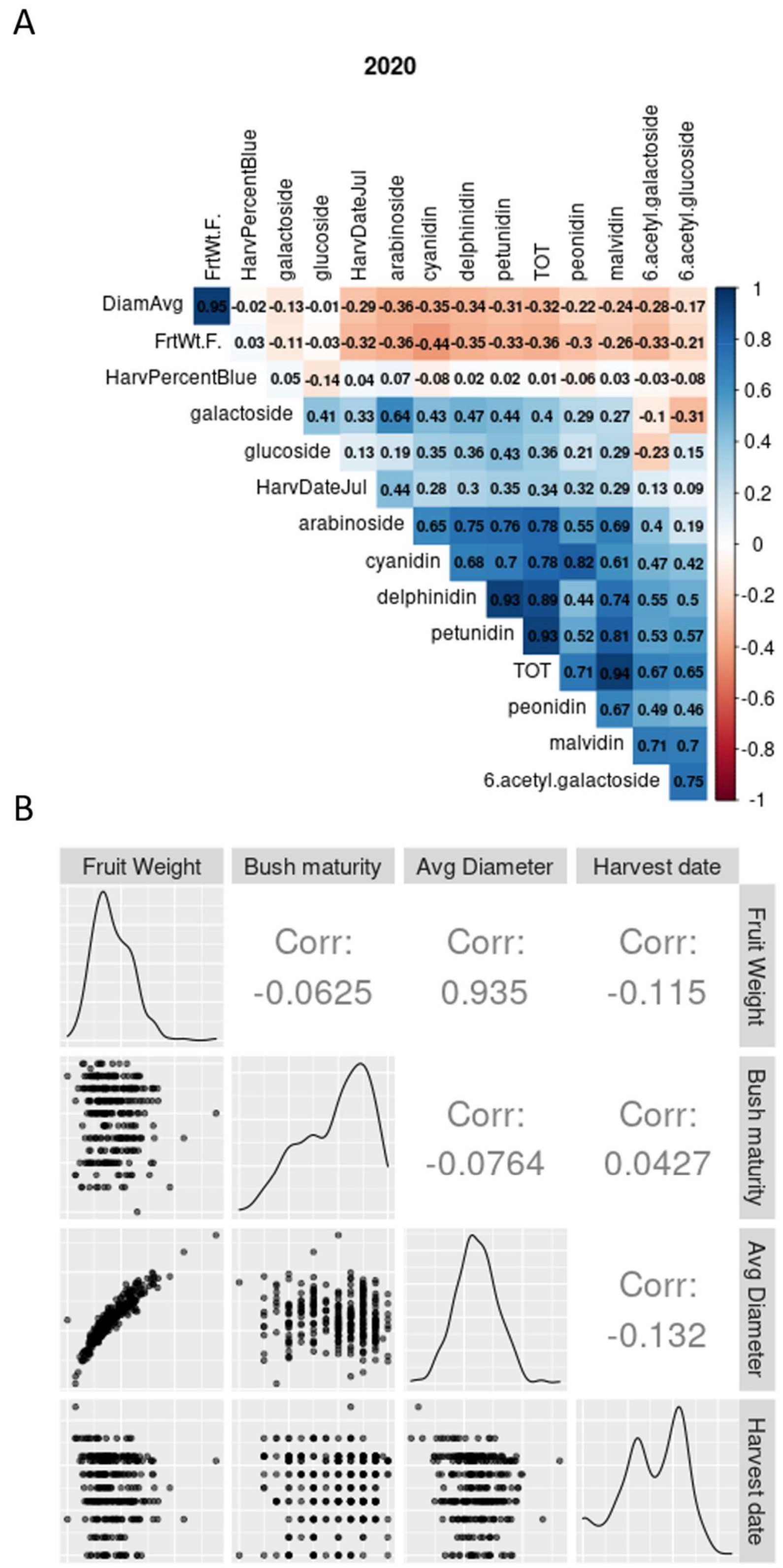

Supplementary Figure 5. Correlation plots among phenotypic data collected in 2020 on 'Hortblue Petite' × 'Nui' population. A) Pairwise correlations among total anthocyanin

concentrations (μg/g), fruit diameter (DiamAvg), fruit weight (FrtWt.F), harvest date (HarvDateJul) and bush maturity (HarvPercentBlue). B) Pairwise correlations among fruit weight, bush maturity, fruit diameter (avg Diameter) and harvest date. The upper panels show correlation coefficients; the diagonal panels show density plots; the lower panel show scatter plots.

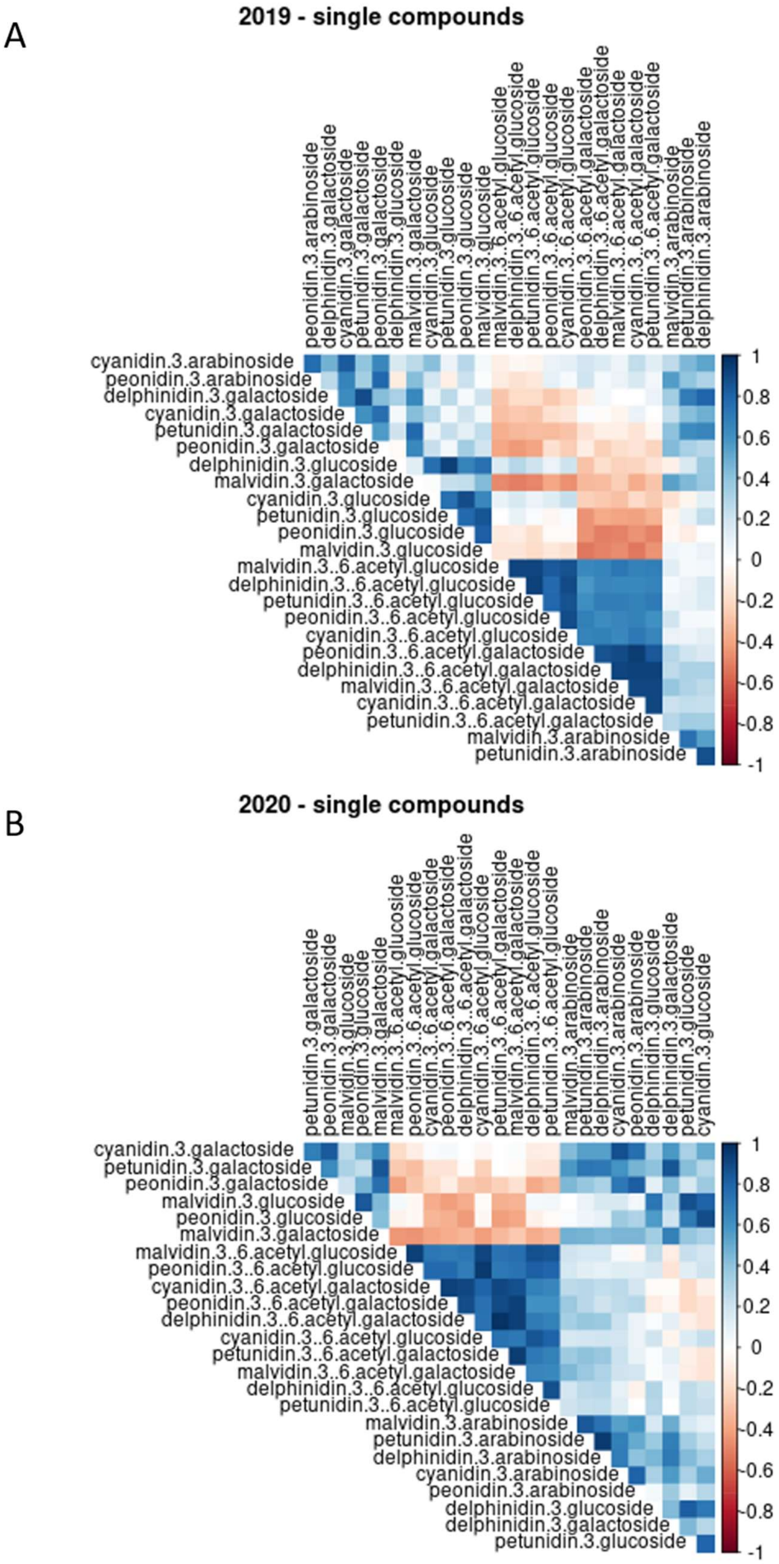

Supplementary Figure 6. Correlation plots among single anthocyanin compounds in ‘Hortblue Petite’ × ‘Nui’ population. For both 2019 (A) and 2020 (B), raw data (anthocyanin concentrations in μg/g) were used.

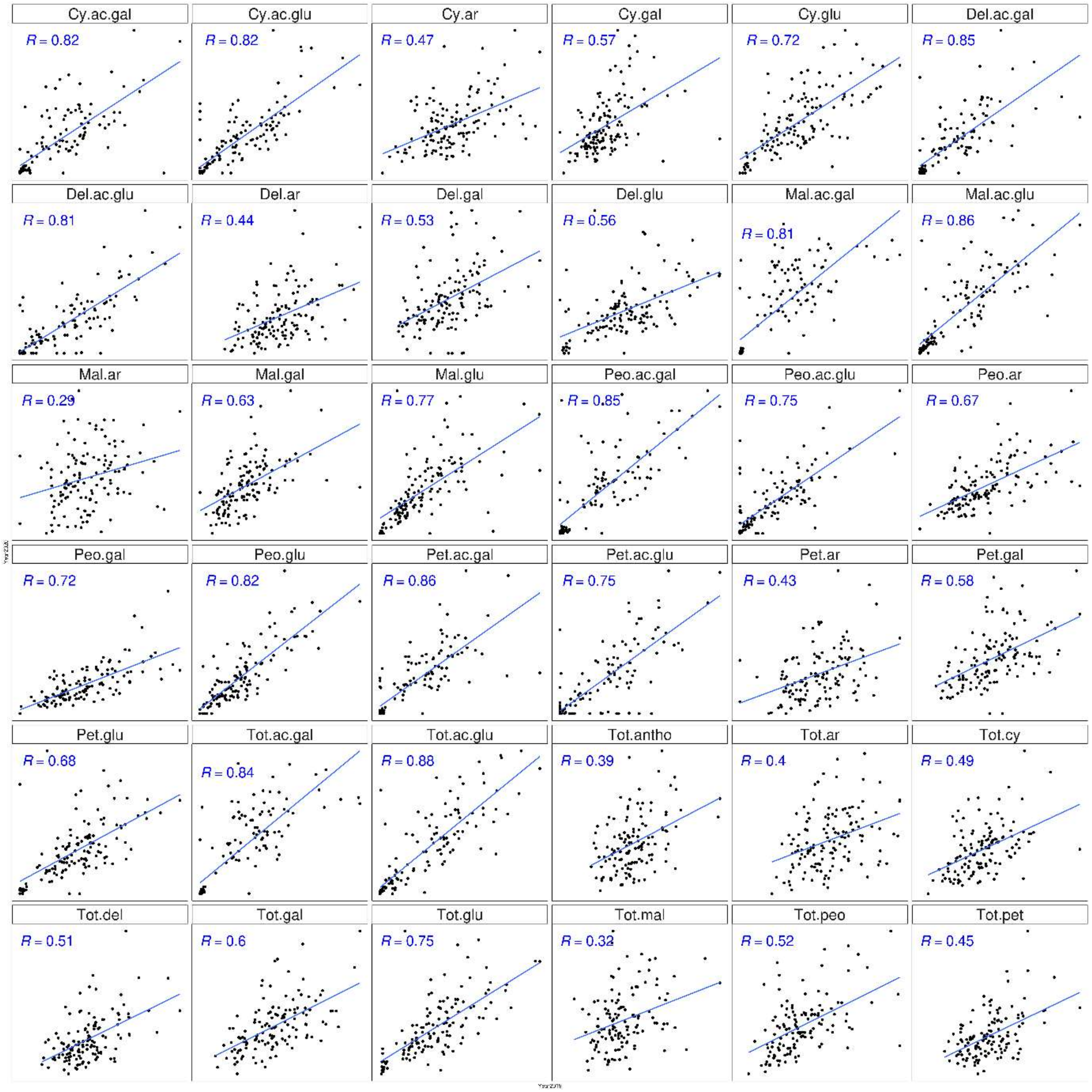

Supplementary Figure 7. Scatter plots showing correlation between two years of phenotyping for anthocyanin content in ‘Hortblue Petite’ × ‘Nui’ population. For each anthocyanin compound and totals, scatter plots between 2019 (x axis) and 2020 (y axis) raw data (anthocyanin concentrations in μg/g) are displayed. Spearman correlation coefficients are reported in blue. cy = cyanidin; del = delphinidin; mal = malvidin; peo = peonidin; pet = petunidin; ac = acetyl; gal = galactoside; glu = glucoside; ar = arabinoside; tot = total

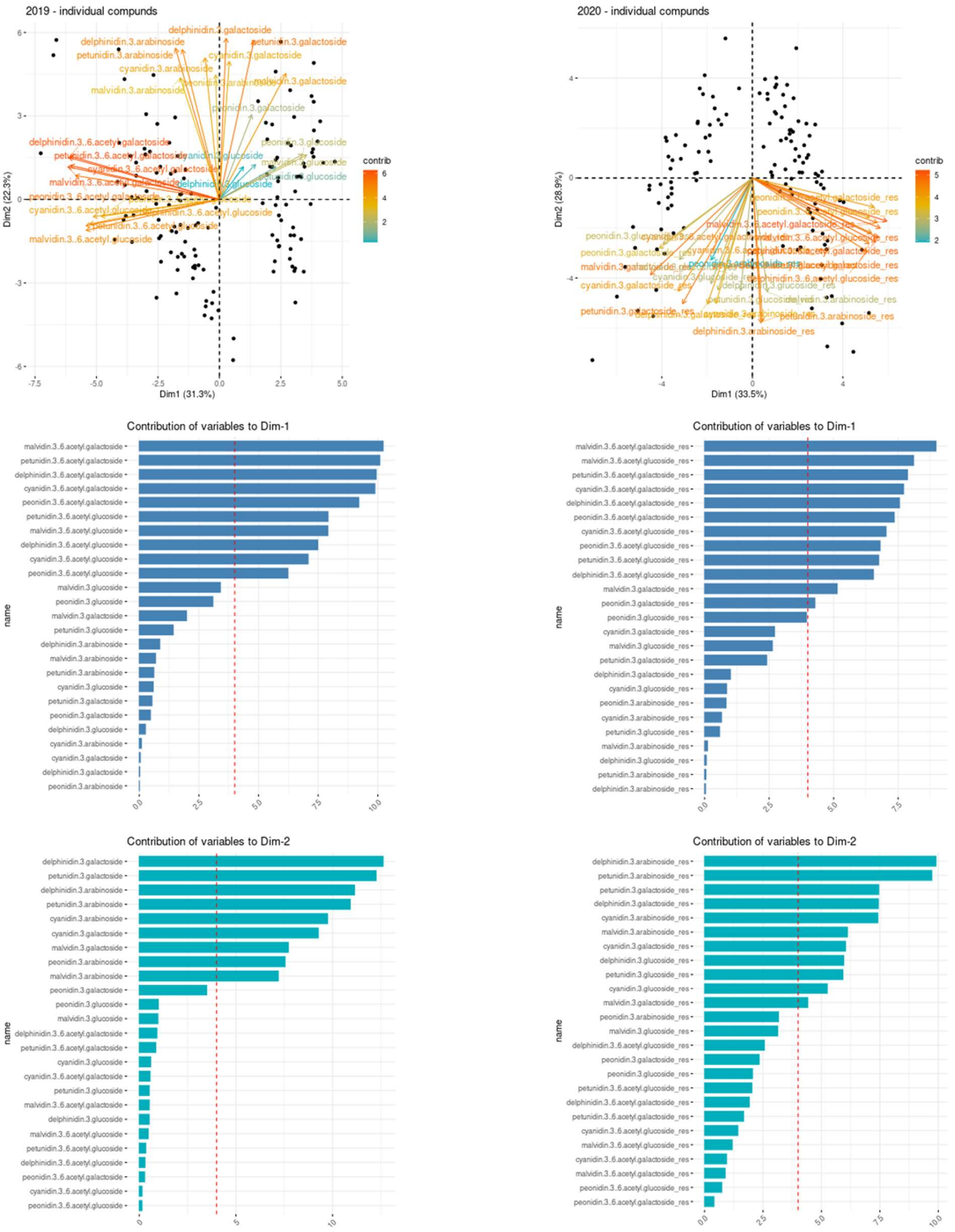

Supplementary Figure 8. Principal Component Analysis (PCA) of single anthocyanin concentrations (μg/g) from 'Hortblue Petite' × 'Nui' population in years 2019 and 2020. For each year, a PC1 versus PC2 plot is shown, as well as the variable contribution (%) to the first and second PC.

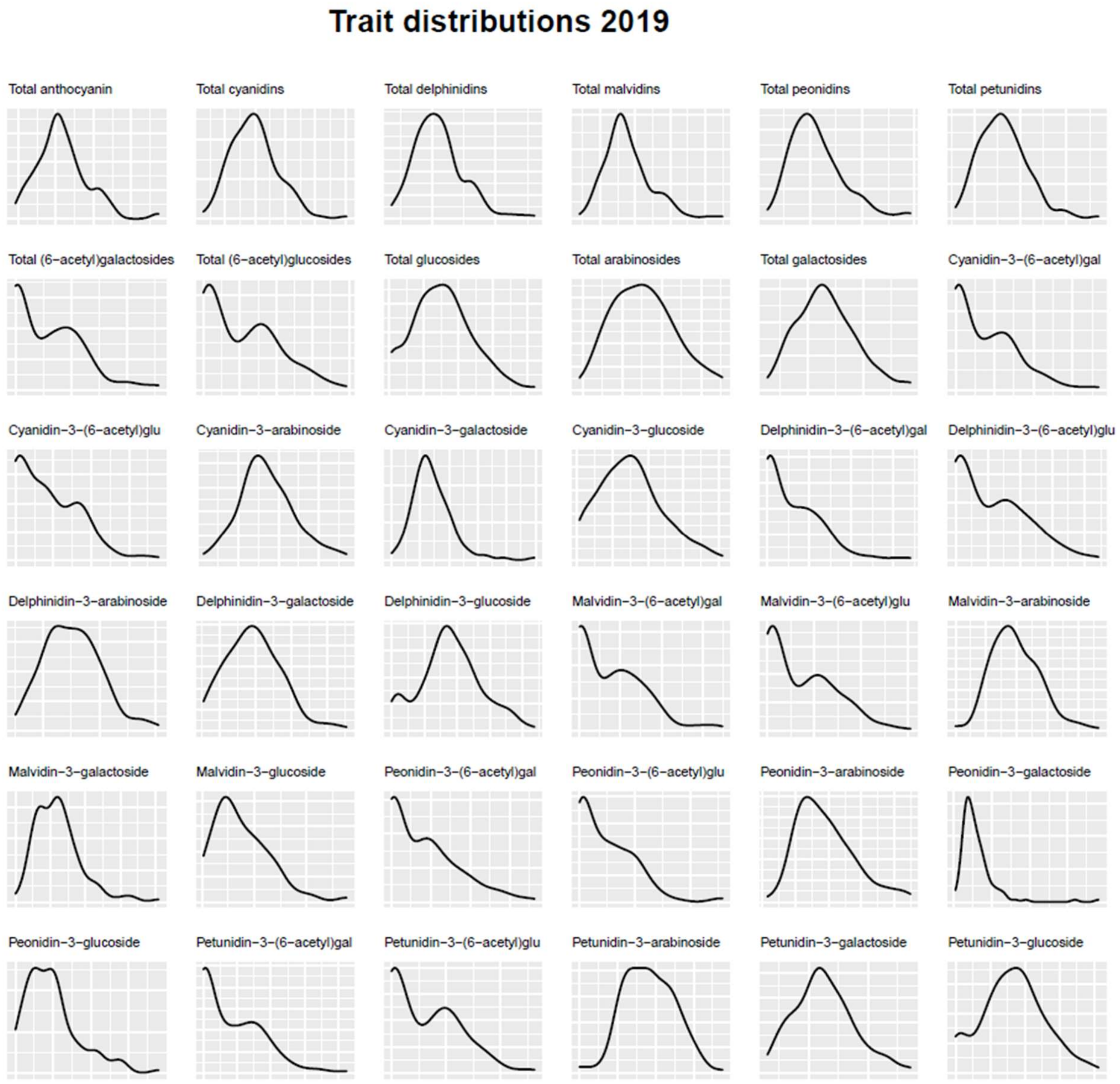
Trait distributions 2019
Total anthocyanin
Total cyanidins
Total delphinidins
Total malvidins
Total peonidins
Total petunidins
Total (6–acetyl)galactosides
Total (6–acetyl)glucosides
Total glucosides
Total arabinosides
Total galactosides
Cyanidin–3–(6–acetyl)gal
Cyanidin–3–(6–acetyl)glu
Cyanidin–3–arabinoside
Cyanidin–3–galactoside
Cyanidin–3–glucoside
Delphinidin–3–(6–acetyl)gal
Delphinidin–3–(6–acetyl)glu
Delphinidin–3–arabinoside
Delphinidin–3–galactoside
Delphinidin–3–glucoside
Malvidin–3–(6–acetyl)gal
Malvidin–3–(6–acetyl)glu
Malvidin–3–arabinoside
Malvidin–3–galactoside
Malvidin–3–glucoside
Peonidin–3–(6–acetyl)gal
Peonidin–3–(6–acetyl)glu
Peonidin–3–arabinoside
Peonidin–3–galactoside
Peonidin–3–glucoside
Petunidin–3–(6–acetyl)gal
Petunidin–3–(6–acetyl)glu
Petunidin–3–arabinoside
Petunidin–3–galactoside
Petunidin–3–glucoside

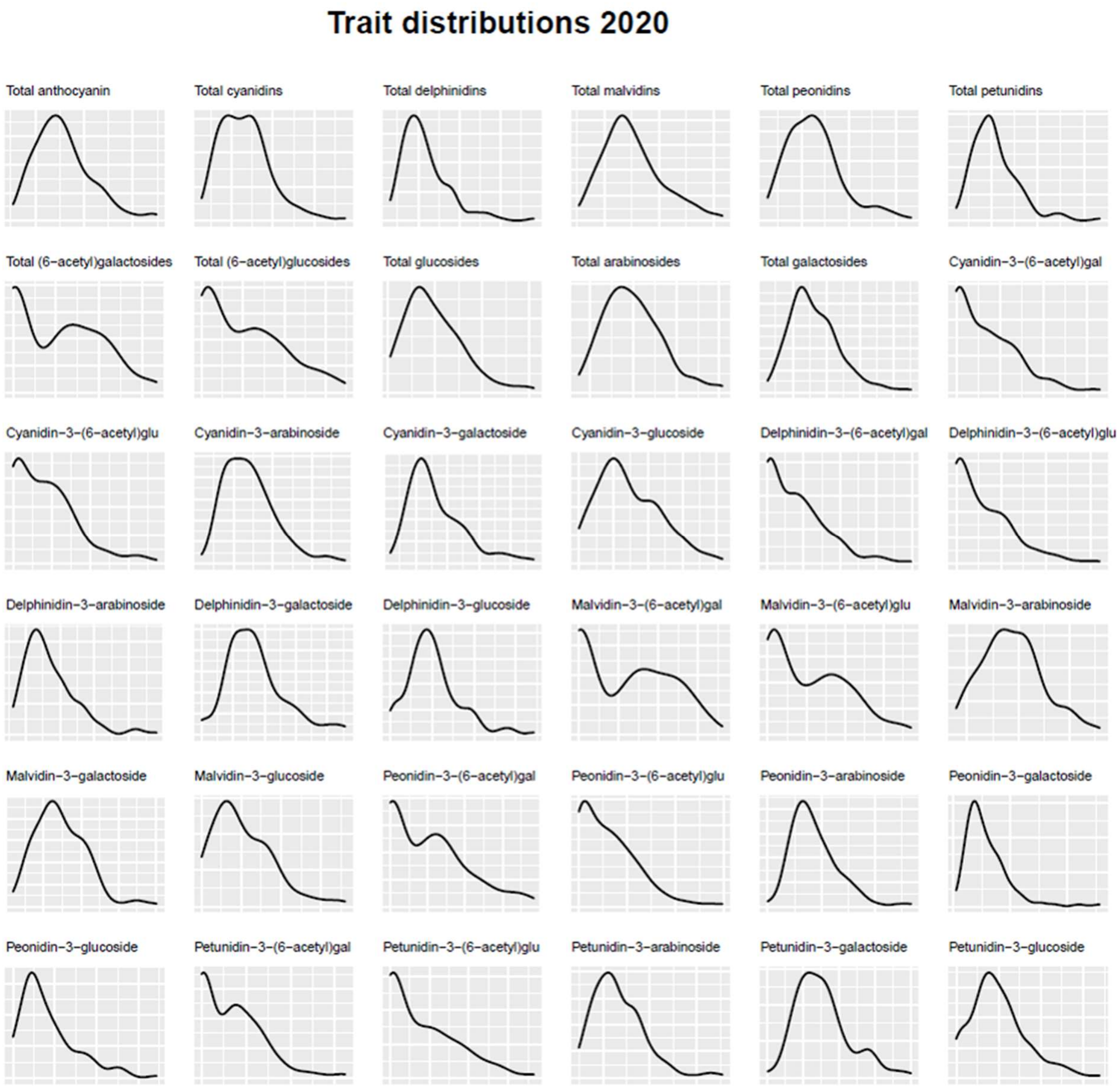
Trait distributions 2020
Total anthocyanin
Total cyanidins
Total delphinidins
Total malvidins
Total peonidins
Total petunidins
Total (6−acetyl)galactosides
Total (6−acetyl)glucosides
Total glucosides
Total arabinosides
Total galactosides
Cyanidin−3−(6−acetyl)gal
Cyanidin−3−(6−acetyl)glu
Cyanidin−3−arabinoside
Cyanidin−3−galactoside
Cyanidin−3−glucoside
Delphinidin−3−(6−acetyl)gal
Delphinidin−3−(6−acetyl)glu
Delphinidin−3−arabinoside
Delphinidin−3−galactoside
Delphinidin−3−glucoside
Malvidin−3−(6−acetyl)gal
Malvidin−3−(6−acetyl)glu
Malvidin−3−arabinoside
Malvidin−3−galactoside
Malvidin−3−glucoside
Peonidin−3−(6−acetyl)gal
Peonidin−3−(6−acetyl)glu
Peonidin−3−arabinoside
Peonidin−3−galactoside
Peonidin−3−glucoside
Petunidin−3−(6−acetyl)gal
Petunidin−3−(6−acetyl)glu
Petunidin−3−arabinoside
Petunidin−3−galactoside
Petunidin−3−glucoside

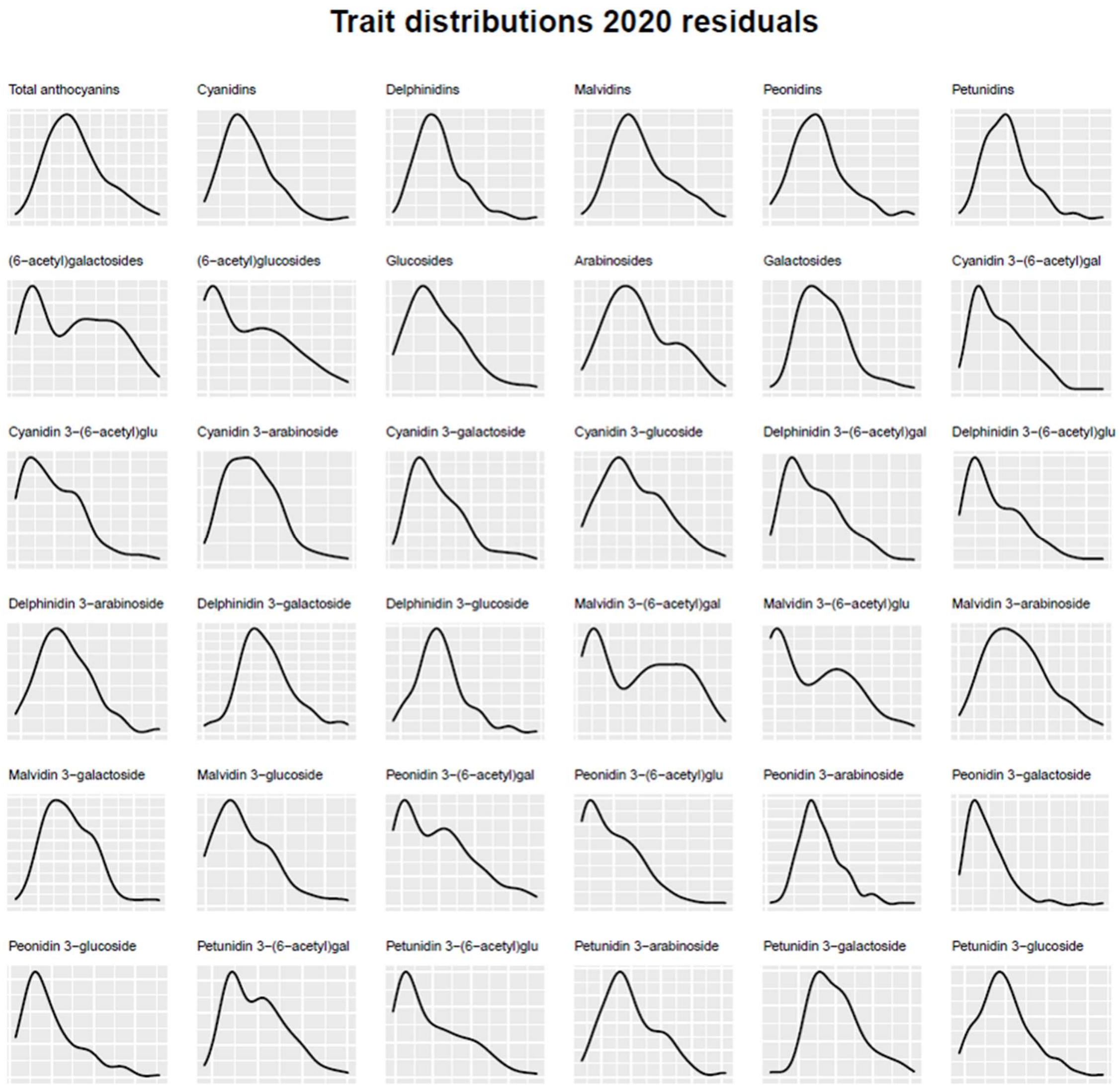

Supplementary Figure 9. Density plots for each trait in 2019 and 2020. For both years, raw data (anthocyanin concentrations in μg/g) were used. For 2020, also distribution of phenotypes adjusted for harvest data (residuals of fitted models) are shown.

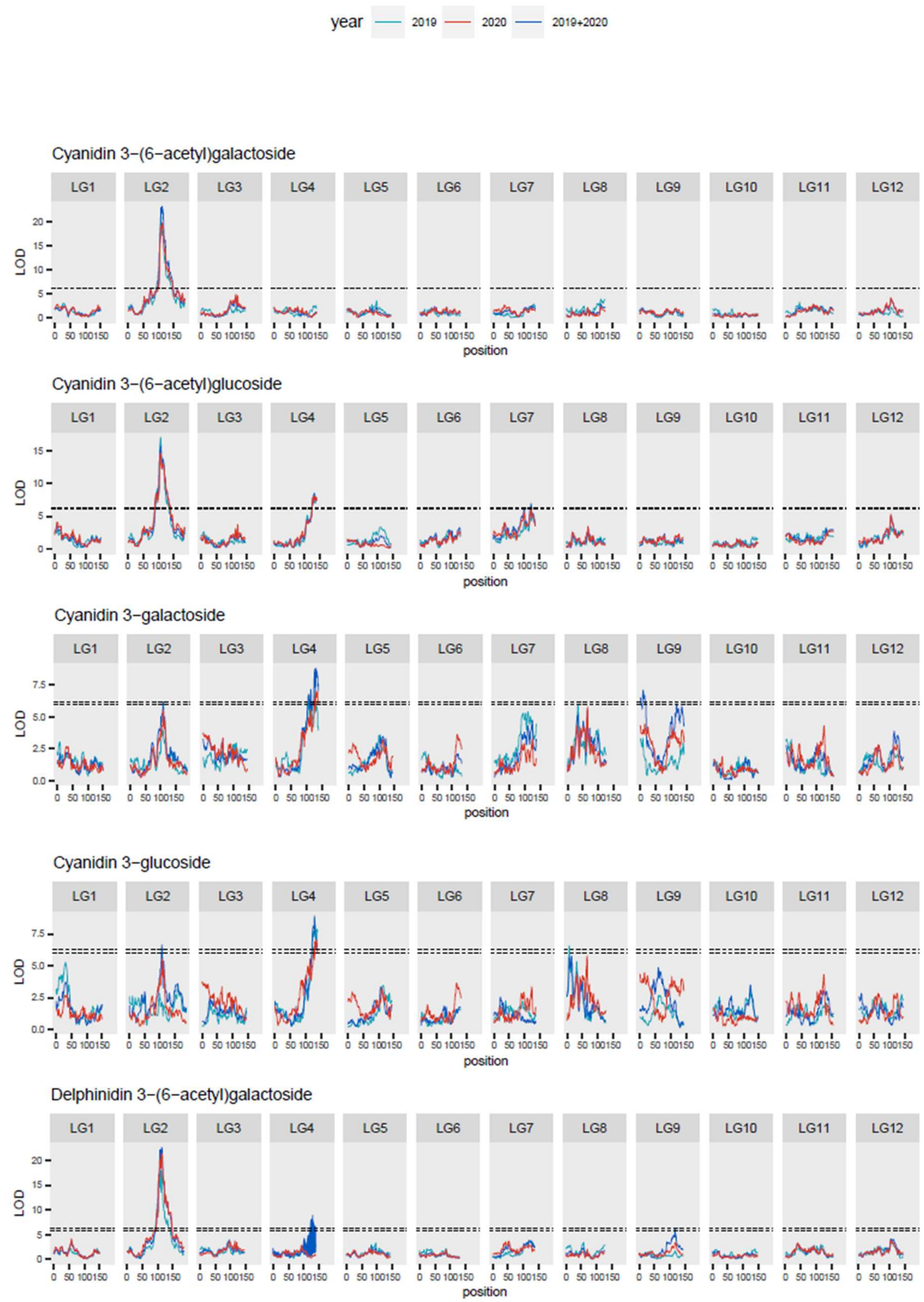
year
2019
2020
2019+2020
Cyanidin 3−(6−acetyl)galactoside
Cyanidin 3−(6−acetyl)glucoside
Cyanidin 3−galactoside
Cyanidin 3−glucoside
Delphinidin 3−(6−acetyl)galactoside
LG1
LG2
LG3
LG4
LG5
LG6
LG7
LG8
LG9
LG10
LG11
LG12
LOD
position

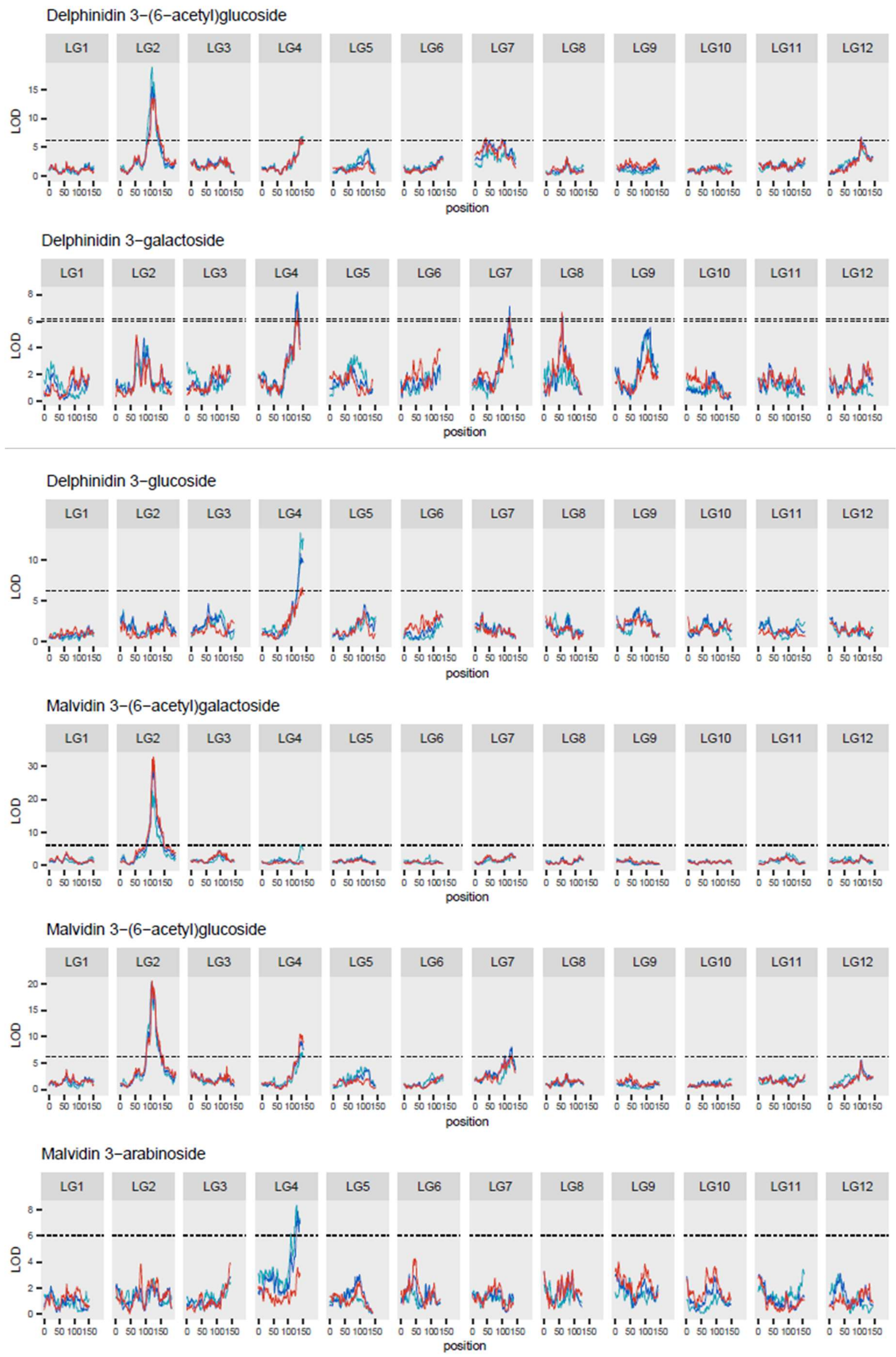
Delphinidin 3−(6−acetyl)glucoside
Delphinidin 3−galactoside
Delphinidin 3−glucoside
Malvidin 3−(6−acetyl)galactoside
Malvidin 3−(6−acetyl)glucoside
Malvidin 3−arabinoside
LG1
LG2
LG3
LG4
LG5
LG6
LG7
LG8
LG9
LG10
LG11
LG12
LOD
position

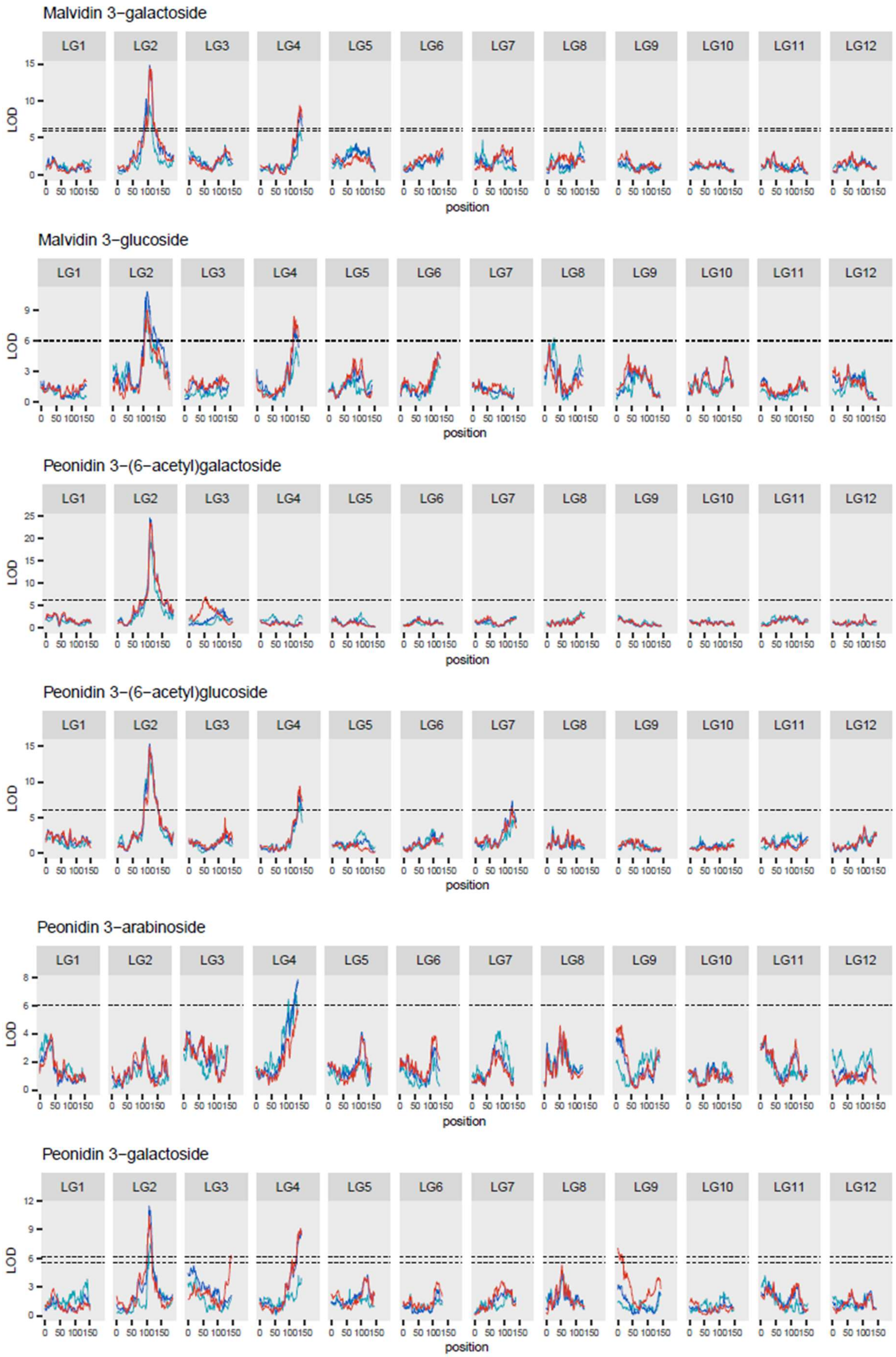
Malvidin 3−galactoside
Malvidin 3−glucoside
Peonidin 3−(6−acetyl)galactoside
Peonidin 3−(6−acetyl)glucoside
Peonidin 3−arabinoside
Peonidin 3−galactoside
LG1 LG2 LG3 LG4 LG5 LG6 LG7 LG8 LG9 LG10 LG11 LG12
LOD
position
0 50 100150

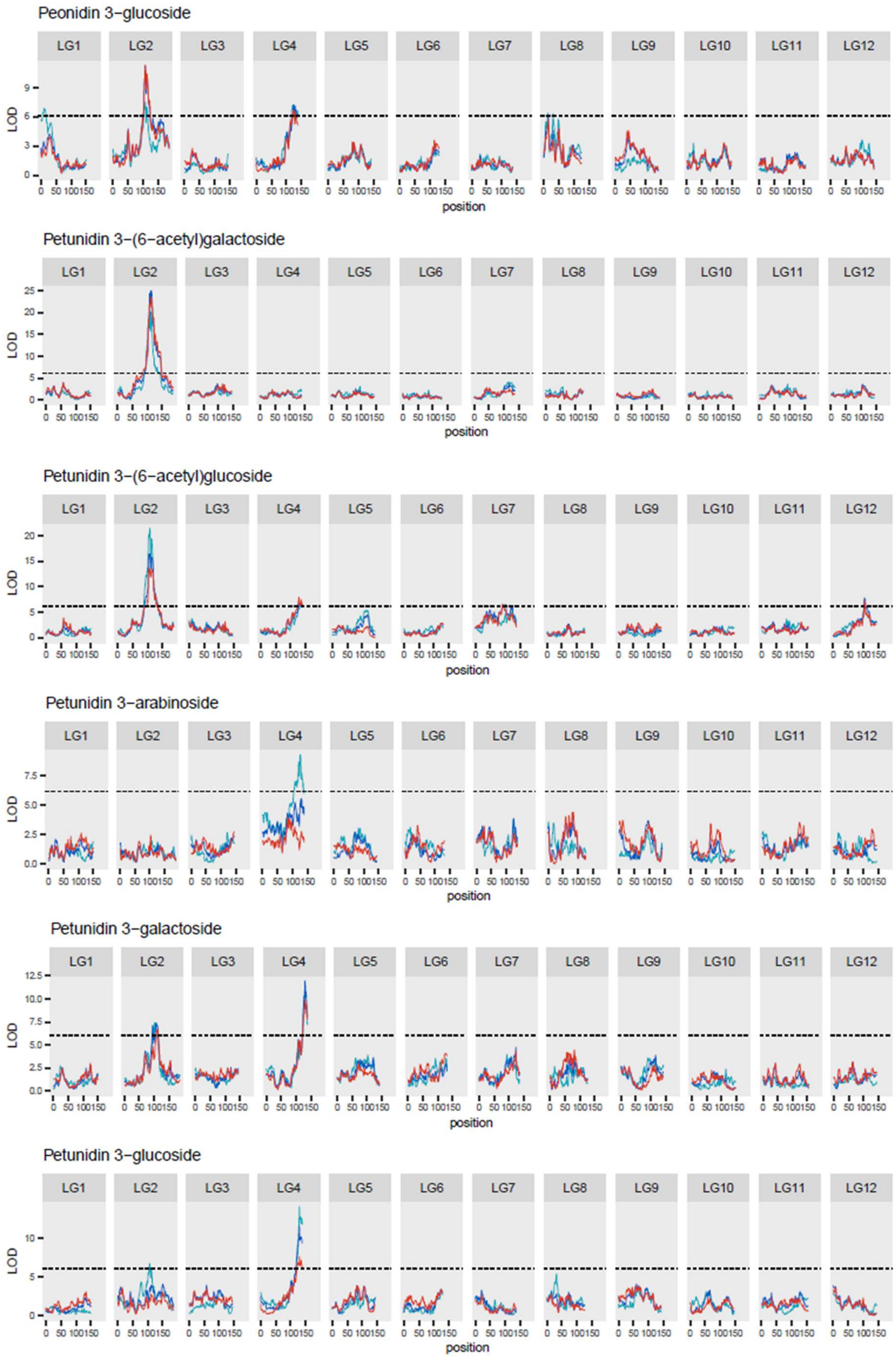
Peonidin 3−glucoside
Petunidin 3−(6−acetyl)galactoside
Petunidin 3−(6−acetyl)glucoside
Petunidin 3−arabinoside
Petunidin 3−galactoside
Petunidin 3−glucoside
LG1
LG2
LG3
LG4
LG5
LG6
LG7
LG8
LG9
LG10
LG11
LG12
LOD
position
0 50 100150

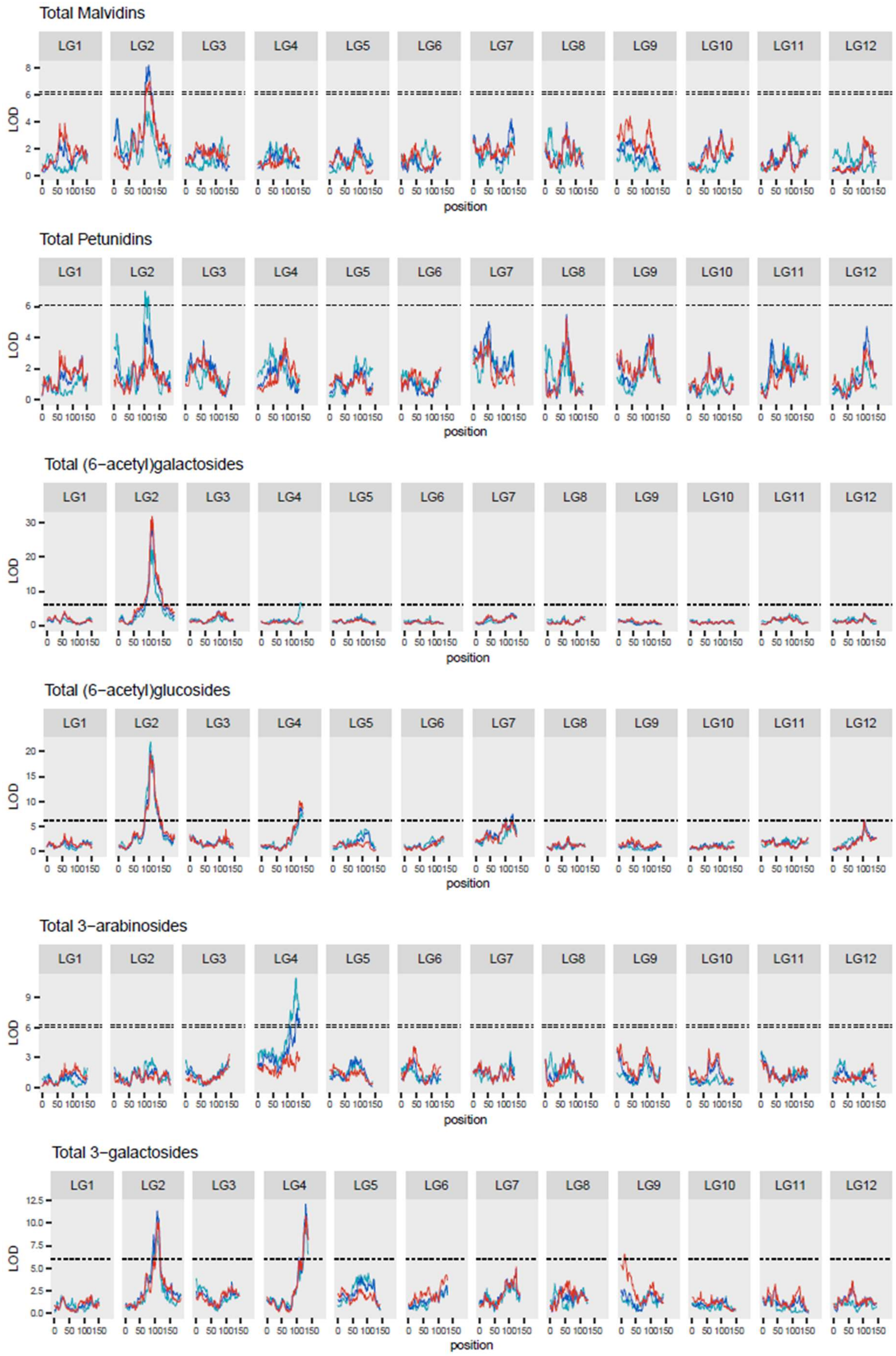
Total Malvidins
Total Petunidins
Total (6−acetyl)galactosides
Total (6−acetyl)glucosides
Total 3−arabinosides
Total 3−galactosides
LG1
LG2
LG3
LG4
LG5
LG6
LG7
LG8
LG9
LG10
LG11
LG12
LOD
position

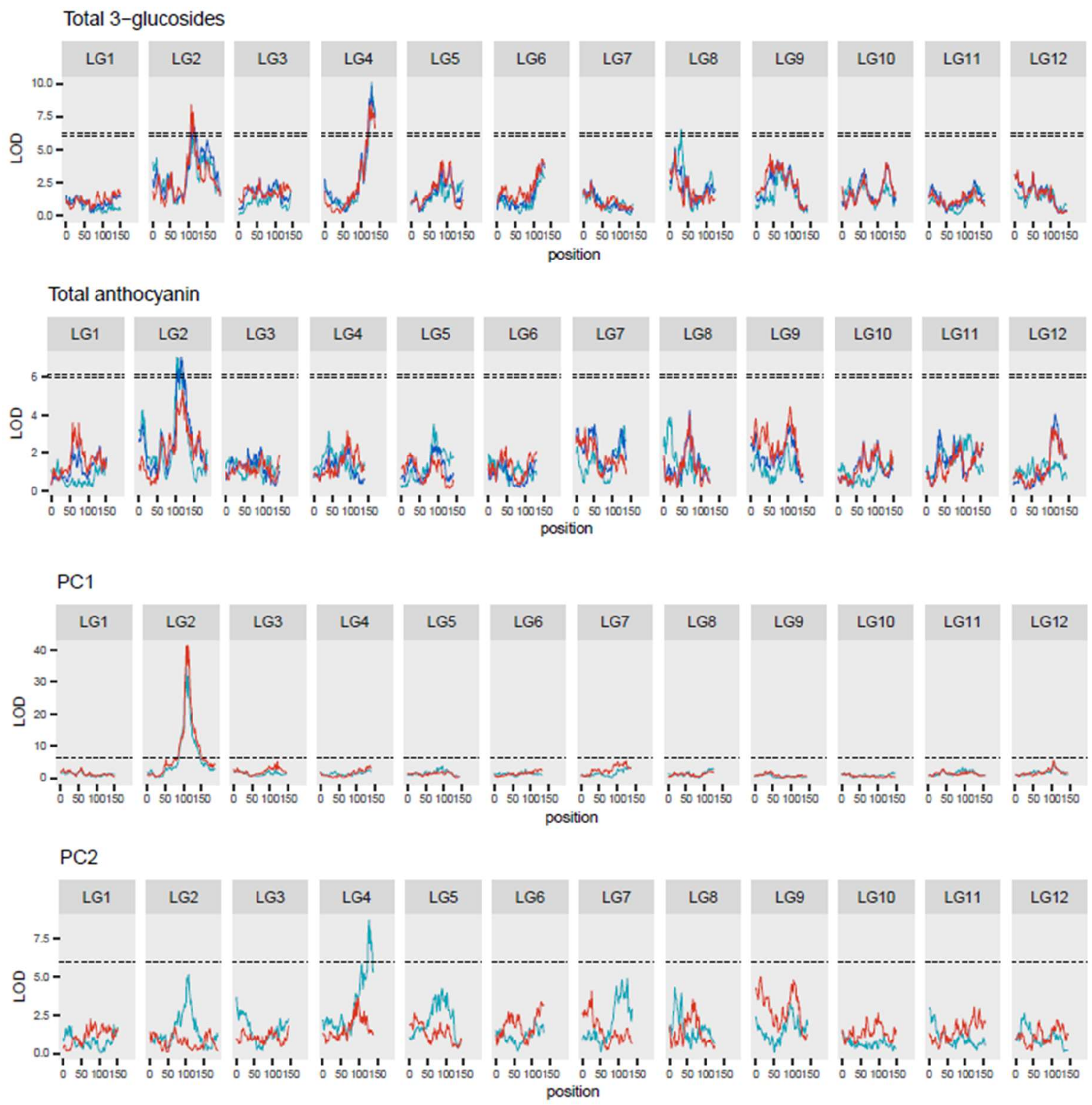

Supplementary Figure 10. Comparison of quantitative trait loci (QTLs) across years and traits. For each anthocyanin trait, the LOD curves on all 12 linkage groups (LGs) are shown for 2019, 2020 and for the two-year combined data.
